# Defining and Measuring Resilience in Children with a Chronic Disease: a Scoping Review

**DOI:** 10.1007/s42844-023-00092-2

**Published:** 2023-04-10

**Authors:** Sabine E. I. van der Laan, Emma E. Berkelbach van der Sprenkel, Virissa C. Lenters, Catrin Finkenauer, Cornelis K. van der Ent, Sanne L. Nijhof

**Affiliations:** 1 Department of Pediatric Pulmonology,Wilhelmina Children’s Hospital, University Medical Center Utrecht, Utrecht University, Utrecht, The Netherlands; 2 Julius Center for Health Sciences and Primary Care, University Medical Center Utrecht, Utrecht University, Utrecht, The Netherlands; 3 Department of Pediatrics, Wilhelmina Children’s Hospital, University Medical Center Utrecht, Utrecht University, Utrecht, The Netherlands; 4 Department of Interdisciplinary Social Sciences, Utrecht University, Utrecht, The Netherlands

**Keywords:** Resilience, Children/adolescent, Chronic disease, Measurement, Definition

## Abstract

More than 25% of all children grow up with a chronic disease. They are at higher risk for developmental and psychosocial problems. However, children who function resiliently manage to adapt positively to these challenges. We aim to systematically review how resilience is defined and measured in children with a chronic disease. A search of PubMed, Cochrane, Embase, and PsycINFO was performed on December 9, 2022, using resilience, disease, and child/adolescent as search terms. Two reviewers independently screened articles for inclusion according to predefined criteria. Extraction domains included study characteristics, definition, and instruments assessing resilience outcomes, and resilience factors. Fifty-five out of 8766 articles were identified as relevant. In general, resilience was characterized as positive adaptation to adversity. The included studies assessed resilience by the outcomes of positive adaptation, or by resilience factors, or both. We categorized the assessed resilience outcomes into three groups: personal traits, psychosocial functioning, and disease-related outcomes. Moreover, myriad of resilience factors were measured, which were grouped into internal resilience factors (cognitive, social, and emotional competence factors), disease-related factors, and external factors (caregiver factors, social factors, and contextual factors). Our scoping review provides insight into the definitions and instruments used to measure resilience in children with a chronic disease. More knowledge is needed on which resilience factors are related to positive adaptation in specific illness-related challenges, which underlying mechanisms are responsible for this positive adaptation, and how these underlying mechanisms interact with one another.

**Supplementary Information:**

The online version contains supplementary material available at 10.1007/s42844-023-00092-2.

## Introduction

The diagnosis and treatment of childhood diseases have advanced significantly in recent decades, which have also led to an improved life expectancy among children with a chronic disease (Mokkink et al., 2008). Recent studies showed that more than 25% of children and young adults under the age of twenty-five suffer from a chronic disease, both in the Netherlands as well as in the USA (van Cleave et al., 2010; van Hal et al., 2019). In light of the increasing prevalence of chronic diseases, Huber (2011) proposed to change the concept of health from “a state of complete physical, mental and social well-being” (World Health Organisation, 2020) to a new dynamic approach focusing more on disease management than pathology, namely “the ability to adapt and self-manage in the face of social, physical and emotional challenges” (Huber et al., 2011).

It has been shown that children with a chronic disease suffer from more physical as well as psychosocial challenges due to symptom distress, demanding therapeutic regimens, periods of hospitalization, uncertainty about the future, social exclusion, and the inability to fully participate in school or society (Compas et al., 2012; Michaud et al., 2004; Perfect & Frye, 2014). However, there are large inter-individual differences and not all children with a similar chronic disease experience (similar) difficulties. While some children do not adapt or even adapt negatively and develop more serious problems, many children manage to positively adapt to these challenges. This phenomenon, “positive adaptation within the context of significant adversity by maintaining or regaining mental health or psychosocial functioning,” is often referred to as resilience (Kalisch et al., 2017; Luthar et al., 2000; Masten, 2011; Oles, 2015). Different concepts of resilience are described, such as — but not limited to — physical resilience and psychological resilience. Physical resilience is often thought of as “the ability to physically recover or optimize function in the face of disease or age-related losses” (Whitson et al., 2016). As clinician-scientists, our research focus is on identifying the factors that contribute to differences in functioning among children with chronic diseases. Specifically, we aim to investigate why some children with the same chronic disease are able to adapt and integrate into society, while others experience difficulties in this regard. Thus, in this review, our focus is on the psychological aspect of resilience, which we refer to as “resilience” throughout this paper. 

In pediatric healthcare, increased awareness of the importance of positive adaptation to stress has led to an increased focus on resilience research (Hilliard et al., 2015). Resilience is a complex concept and various resilience frameworks have been developed to clarify the concept (Hilliard et al., 2015). As a result, definitions and instruments used to assess resilience in pediatric healthcare research vary greatly between studies and might lead to lack of clarity within the field (Hilliard et al., 2015; Perfect & Frye, 2014). Some investigators defined and measured resilience as an outcome by assessing outcomes of positive adaptation to adversity (i.e., disease), for instance, in terms of psychosocial functioning (e.g., mental wellbeing, QoL, lack of mental health problems, and cognitive abilities) (Breda, 2018). Others tried to explain why individuals are able to positively adapt and maintain good mental wellbeing and therefore focus on the factors that facilitate positive adaptation to adversity. These factors are generally referred to as resilience factors (Breda, 2018; Kalisch et al., 2017). These resilience factors can be roughly divided into internal factors and external factors. The first group consists of factors that can be related to the child’s biology but also to the child’s behavior, emotions, and cognition (Fritz et al., 2018; Ioannidis et al., 2020). External factors relate not only to various aspects of the child’s environment, such as relationships with parents and friends (Afifi & MacMillan, 2011; Rutter, 1985; van Harmelen et al., 2017), but also contextual factors such as educational, and cultural environment (Liebenberg et al., 2012).

The aim of the present review is to provide an overview of definitions and instruments used to assess resilience in children with a chronic disease. Hereby, we seek to identify commonalities and differences between these definitions of childhood resilience. Moreover, we provide an overview of resilience *outcomes* and resilience *factors* in the field of pediatric care. Identifying these outcomes and factors can improve the care of children with chronic illness by providing insights for interventions and preventive strategies aimed at adapting best to the challenges posed by chronic illness.

## Methods

### Search Strategy

A systematic review of the available literature was conducted on December 9, 2022, according to the Preferred Reporting Items for Systematic Reviews and Meta-analyses (PRISMA) Extension for Scoping Reviews (Tricco et al., 2018). An electronic search of PubMed, Cochrane, Embase, and PsycINFO was performed to identify relevant peer-reviewed articles. The search terms included resilience, (chronic) disease, and child/adolescent — in combination with the corresponding Medical Subject Headings (MeSH) terms and synonyms. The complete search strings were approved by a medical librarian at the University Medical Centre Utrecht and can be found in Supplement 1. This review is registered in PROSPERO, the international prospective register of systematic reviews (ID:147023).

### Selection Criteria

Our pre-defined study selection criteria were the following: (1) pediatric sample; (2) having a physical chronic disease; (3) with self-reported measurement(s) of resilience; (4) published in English in a peer-reviewed journal; and (5) available in full text. Exclusion criteria were: (1) articles that mentioned resilience in the text but did not measure resilience; (2) articles in which resilience was merely defined as “not having a disease;” (3) parental-reported resilience; (4) qualitative research (e.g., interviews); (5) studies with only a limited number of participants (e.g., case-reports); (6) studies that provide too little or no details on the quantitative methods and/or results (conference abstracts, protocols); (7) articles that focused on children with medically unexplained symptoms; (8) articles that focused on children with psychiatric disorders; (9) articles that used the exact same study population as previously studied articles (in that case, we retained the first published article); (10) studies validating a resilience instrument; and (11) articles that focused on resilience related to the coronavirus disease-2019 (COVID-19) pandemic among children with a chronic disease instead of resilience of children with a chronic disease. There was no restriction in terms of publication date of the included articles.

A pediatric chronic disease was defined as “a condition that occurs between the age of 0–18 years, was diagnosed by a professional based on medical scientific knowledge using valid methods and instruments, is not (yet) curable and has existed for more than three months” (Mokkink et al., 2008).

We only included self-reported measurements of resilience, as children and their parents or caregivers may experience certain (internal) resilience factors in a different way. For example, research shows that parents overestimate their children’s optimism and underestimate their worries.(Lagattuta et al., 2012) When included studies used proxy-reported or qualitative instruments (besides self-reported measurements of resilience), we did not present these in our overviews (Table 1; Fig. 2; Supplement 3; Fig. 3; and Supplement 4). Some studies used blood tests to measure metabolic control (as a resilience outcome) next to self-reported questionnaire methods (Jaser & White, 2011; LeBovidge et al., 2009; F. R. M. Santos et al., 2013; Yi-Frazier et al., 2015); we also presented the blood test, as this test is a quantitative instrument.

### Study Selection

All identified articles were uploaded in Rayyan QCRI, a web-based tool, in which titles and abstracts were independently reviewed by two researchers (SvdL and EBvdS). Each researcher assigned the article to one of three categories: include, exclude, or maybe. Articles with labels “include” and “maybe” were selected for full-text screening. Those articles were evaluated for eligibility according to the inclusion and exclusion criteria, again independently by the same two researchers (SvdL and EBvdS). In 85% of all included articles, the researchers agreed that the articles met the inclusion criteria. In the remaining 15%, a consensus was reached through discussion.

### Article Review and Data Extraction

For each article, the following characteristics were extracted: first author, year of publication, diagnosis, sample size, age of study population, country, study design, definition of resilience (when provided), and instruments measuring resilience outcomes and/or resilience factors (see Supplement 2)*.* To further examine the instruments used by the included articles, the following characteristics of the instruments were extracted: items, response, and range (Supplements 3 and 4). A risk of bias assessment was not performed, as the findings of the individual studies were not the primary interest of this review.

Figures 2 and 3 presented in this study are created using Datylon, a data visualization software (Datylon, 2023).

## Results

### Search and Baseline Characteristics

**Fig. 1:**
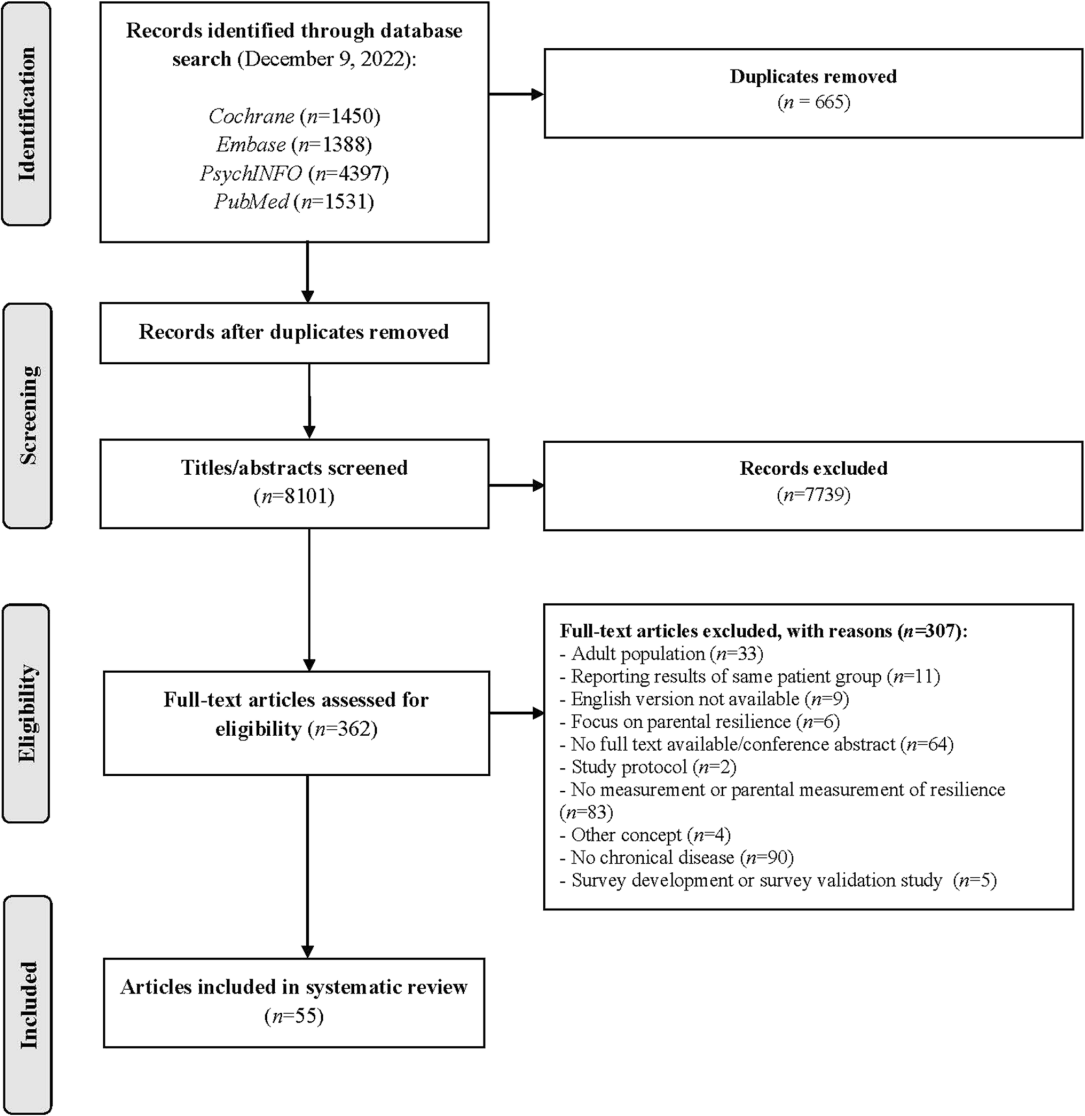
PRISMA flow diagram of selection process

A total of 8766 articles were identified through the literature search. After removal of duplicates, the remaining 8101 articles were uploaded to Rayyan QCRI (Rayyan, 2019). A total of 362 articles were identified for full text review and evaluated according to the inclusion criteria. In total, 55 articles were included. The flow diagram is shown in Fig. 1*.*

The articles represented study populations from fourteen different countries, and 25 (45%) studies were conducted in the USA. The articles were published between 2009 and 2022 and encompassed the following chronic diseases: malignancies (*n* = 12), type 1 or type 2 diabetes (*n* = 8), atopic diseases (*n* = 4), non-malignant neurological diseases (*n* = 5), congenital heart disease (CHD) (*n* = 4), auto-immune disorders (*n* = 4), non-malignant hematological diseases (*n* = 4), human immunodeficiency virus/acquired immune deficiency syndrome (*n* = 2), obesity (*n* = 2), otolaryngology problems (*n* = 2) or, other or combination of the aforementioned (*n* = 8) (see Supplement 2). Table 1 presents a general definition and specific aspects of resilience summarizing all included definitions. Additionally, Table 1 provides information on how often resilience was measured as an outcome, as resilience factors, or as both. Supplement 2 demonstrates the specific characteristics of all included articles.

### Definition of Resilience in the Context of Childhood Chronic Diseases

Various definitions of the concept of resilience were found in the included articles. In total, 46 (84%) of the articles provided a definition of resilience. Of all definitions, the definition provided by Luthar et al. (2000) was cited most frequently (*n* = 6): “Resilience refers to a dynamic process encompassing positive adaptation within the context of significant adversity” (Luthar et al., 2000). The other definitions (*n* = 40) are presented in Supplement 2. In the presented definitions, resilience was characterized as emerging in times of stress or emergency to ensure maintenance of health, psychological, and/or social well-being and was often considered an adaptation process to a stressor. In total, 15 (27%) articles described resilience as a personal trait or skill, whereas 11 (20%) articles characterized resilience as a multi-dimensional concept. The majority of the included articles (*n* = 29, 53%) did not explicitly mention (or gave no definition at all) whether resilience was seen as a personal trait or multi-dimensional concept (Table 1).

**Table 1:** Definition and measurement of resilience, as presented in the included studies

**Definition of resilience**		
Total included: *n* = 55	No definition provided	9 (16%) (Araia et al., 2020; Huston et al., 2016; LeBovidge et al., 2009; Santos et al., 2016; Schwartz & Brumley, 2017; Sharp et al., 2018; Sharp et al., 2015; Tang et al., 2022; Willard et al., 2018)
	Definition provided	46 (84%) (Adibsereshki et al., 2021; Arruda et al., 2021; Bahryni et al., 2016; Beeckman et al., 2019; Biernacka et al., 2021; Borinsky et al., 2019; Carlsen et al., 2017; Chung et al., 2021; Cui et al., 2022; Fee & Hinton, 2011; Gmuca et al., 2021; Hood et al., 2018; Huang et al., 2018; Im & Kim, 2012; Jaser & White, 2011; Kaewkong et al., 2020; Kim & Im, 2014; Kim & Yoo, 2010; Kully-Martens et al., 2021; Lau et al., 2020; Lee et al., 2019; Lee et al., 2014, 2017, 2020; Li et al., 2022; Lukacs et al., 2018; McGavock et al., 2018;Moreira et al., 2015; Nabors et al., 2021; Parviniannasab et al., 2022; Rainone et al., 2017; Rassart et al., 2016; Robb et al., 2014; Rosenberg et al., 2014, 2018; Ruff et al., 2016; Shapiro et al., 2021; Simon et al., 2009; Tomlinson et al., 2021; Verma & Rohan, 2020; Whiteley et al., 2019; Wright et al., 2021; Wu et al., 2013; Wu et al., 2018; Yi-Frazier et al., 2015; Zimmerman et al., 2021)
*In the presented definitions, resilience was characterized as emerging in times of stress or emergency to ensure maintenance of health and psychological and/or social well-being and was often considered an adaptation process to a stressor*		
**Aspects of resilience definition** *Some examples are described below*		
*Personal trait/skill* *n* = 15 (27%) (Bahryni et al., 2016; Biernacka et al., 2021; Carlsen et al., 2017; Chung et al., 2021; Hood et al., 2018; Huang et al., 2018; Kim & Yoo, 2010; Lee et al., 2019; Parviniannasab et al., 2022; Rosenberg et al., 2014; Simon et al., 2009; Tomlinson et al., 2021; Wright et al., 2021; Wu et al., 2018; Yi-Frazier et al., 2015)	• “The ability to adapt to situations and environments through self-regulation.”(Gheshlagh et al., 2016) • “Resilience is defined as an individual's strength and ability to moderate the negative effects of stress, promote adaptation, and maintain mental well‐being in the face of adversity.”(Wagnild & Young, 1993); (Davydov et al., 2010) • “(…) a universal construct describing an individual’s capacity to maintain psychological and/or physical well-being in the face of stress and is a good candidate to buffer the negative impact of serious illness among multiple populations of adolescents and young adults.” (Haase, 2004); (Southwick & Charney, 2012) • “Psychological resilience is mentioned among the various psychological resources facilitating beneficial adaptation to illness. Resilience is revealed in the context of coping with negative life events and difficulties. In this sense, resilience can be viewed as an indicator of mental strength. Thanks to it, despite adversities, a person can develop and maintain mental health. (…) When it comes to a chronic illness, psychological resilience is a resource that promotes adaptation to the circumstances of the illness and the limitations generated by it.”(Rutter, 2012), (Kim et al., 2019)	
*Multi-dimensional concept* *n* = 11 (20%) (Cui et al., 2022; Fee & Hinton, 2011; Im & Kim, 2012; Lau et al., 2020; Lee et al., 2017; Lukacs et al., 2018; Nabors et al., 2021; Rainone et al., 2017; Rosenberg et al., 2018; Shapiro et al., 2021; Zimmerman et al., 2021)	• “A process of harnessing resources needed to sustain individual well-being.”(Haase et al., 1999; Rosenberg et al., 2014; Southwick et al., 2014) • “(…) “a dynamic process encompassing positive adaptation within the context of significant adversity.” Resilience is ‘the ability to maintain a stable equilibrium’ (…) It is not a particular personality trait but a process by which positive adaptation occurs despite adversity.”(Werner, 1989) • “Resilience refers to the family’s ability to withstand stressful experiences and rebound from them by creating new, healthy ways of functioning.” (Walsh, 2003) • “(…) a capacity of a dynamic system that helps individuals to overcome the negative effects, recover from adverse circumstances while maintaining normal development. It is not a quality that is always present in every situation, but a process of harnessing new and existing resources to maintain well-being during and after any stressor.” (Rosenberg & Yi-Fraizer, 2016; Masten, 2014)	
*Not explicitly mentioned* *n* = 29 (53%) (Adibsereshki et al., 2021; Araia et al., 2020; Arruda et al., 2021; Beeckman et al., 2019; Borinsky et al., 2019; Gmuca et al., 2021; Huston et al., 2016; Jaser & White, 2011; Kaewkong et al., 2020; Kim & Im, 2014; Kully-Martens et al., 2021; LeBovidge et al., 2009; Lee et al., 2014, 2020; Li et al., 2022; McGavock et al., 2018; Moreira et al., 2015; Rassart et al., 2016; Robb et al., 2014; Ruff et al., 2016; Santos et al., 2016; Schwartz & Brumley, 2017; Sharp et al., 2018; Sharp et al., 2015; Tang et al., 2022; Verma & Rohan, 2020; Whiteley et al., 2019; Willard et al., 2018; Wu et al., 2013)	• “A dynamic process encompassing positive adaptation within the context of significant adversity.” (Luthar et al., 2000) • “The ability to function with healthy responses despite the presence of significant stress and adversity.” (Masten & O’Connor, 1988) • “The protective factors that dynamically allow one to have a good outcome, over-coming stress and adversity, while sustaining normal psychological and physical functioning.” (Masten, 2001; Wu et al., 2013) • “A positive psychological adjustment in the face of adversity which is associated with improved health outcomes in patients with chronic conditions.” (Wu et al., 2016)	
**Resilience measured as**		
Outcome, *n* (%)*	Factor, *n* (%)**	Outcome and factor, *n* (%)
12 (22%) (Adibsereshki et al., 2021; Arruda et al., 2021; Kaewkong et al., 2020; Lee et al., 2017, 2020; Moreira et al., 2015; Nabors et al., 2021; Parviniannasab et al., 2022; Rosenberg et al., 2014, 2018; Tang et al., 2022; Zimmerman et al., 2021)	7 (13%) (Biernacka et al., 2021; Cui et al., 2022; Gmuca et al., 2021; Hood et al., 2018; Li et al., 2022; Shapiro et al., 2021; Whiteley et al., 2019)	36 (65%) (Araia et al., 2020; Bahryni et al., 2016; Beeckman et al., 2019; Borinsky et al., 2019; Carlsen et al., 2017; Chung et al., 2021; Fee & Hinton, 2011; Huang et al., 2018; Huston et al., 2016; Im & Kim, 2012; Jaser & White, 2011; D. H. Kim & Im, 2014; Kim & Yoo, 2010; Kully-Martens et al., 2021; Lau et al., 2020; LeBovidge et al., 2009; Lee et al., 2019; Lee et al., 2014; Lukacs et al., 2018; McGavock et al., 2018; Rainone et al., 2017; Rassart et al., 2016; Robb et al., 2014; Ruff et al., 2016; Santos et al., 2016; Schwartz & Brumley, 2017; C. Sharp et al., 2018; Sharp et al., 2015; Simon et al., 2009; Tomlinson et al., 2021; Verma & Rohan, 2020; Willard et al., 2018; Wright et al., 2021; Wu et al., 2013; Wu et al., 2018; Yi-Frazier et al., 2015)

^*^Please see Fig. 2 and Supplement 3

^**^Please see Fig. 3 and Supplement 4

### Measurement of Resilience in Children with a Chronic Disease

**Fig. 2:**
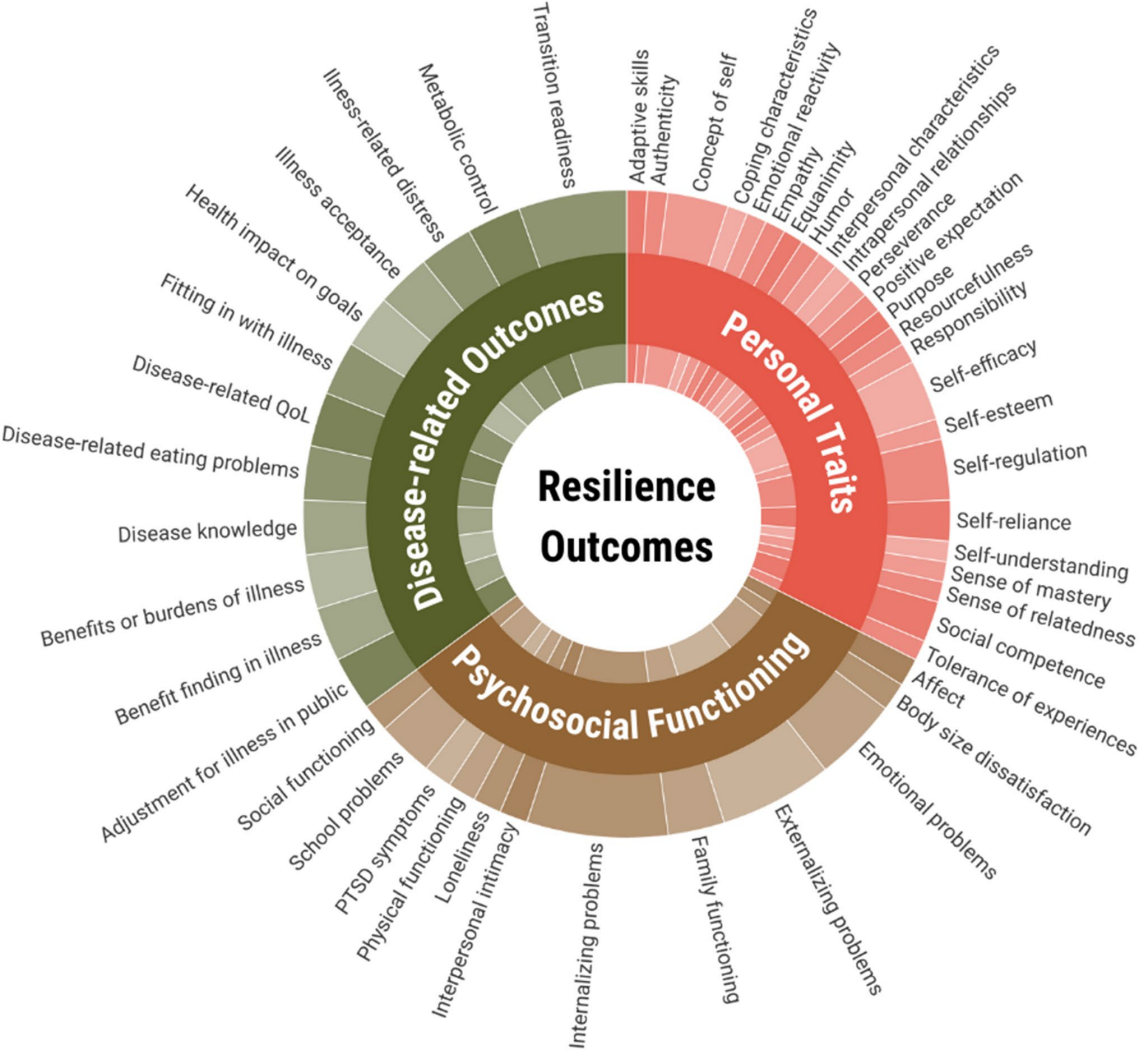
Pie chart illustrating the variety and distribution of resilience outcomes (*n* = 69) measured by 34 instruments in the included studies (see Supplement 3). The chart presents three main categories: personal traits, psychosocial functioning, and disease-related outcomes — with slice and category sizes corresponding to the proportion of total amount of reported resilience outcomes

**Fig. 3:**
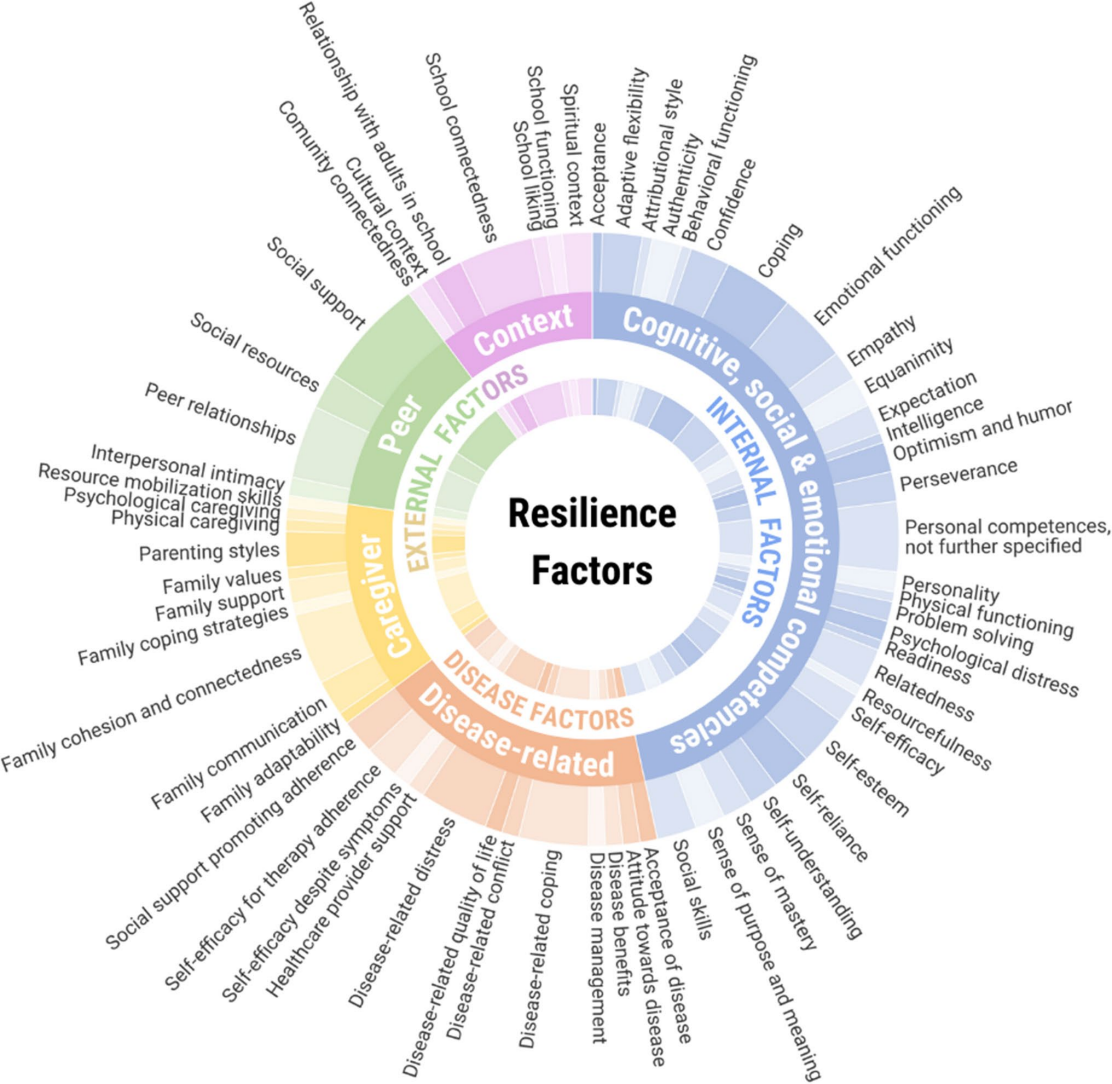
Pie chart illustrating the heterogeneity of resilience factors (*n* = 155) assessed by 66 instruments from the included studies (see Supplement 4). The chart shows three overarching categories: internal, external, and disease-related factors. Internal factors encompassed of cognitive, emotional, and social competence factors. External factors were further classified into caregiver factors, peer factors, and contextual factors. Slice as well as category size indicates the proportion of the total amount of reported resilience factors

In this scoping review, we analyzed which resilience outcomes (Fig. 2; Supplement 3) and which resilience factors (Fig. 3; Supplement 4) were measured in the included articles. To identify the specific topics that were being assessed by each instrument, we reviewed the corresponding or background articles (for references, see Supplements 3 and 4). The topics are then quantified and categorized, and the resultant distribution is depicted in Figs. 2 and 3. It should be noted that not all articles explicitly reported the topics that were assessed by an instrument. To offer a comprehensive overview of the resilience outcomes and factors examined in the literature, we retrieved all themes from the source file and included them in the figures separately.

In total, 36 (65%) studies measured both a resilience outcome as well as resilience factors, and statistically tested whether certain resilience factors were significantly associated with the resilience outcome. For example, Willard et al. (2018) assessed if connectedness to the social environment (such as connectedness to friends and family) influenced social functioning in children with brain tumors (Willard et al., 2018). In this case, the researchers considered social functioning as a resilience outcome and connectedness as resilience factor. Of the remaining 19 studies, 12 (22%) measured only one or more resilience outcome, without assessing resilience factors. For instance, Rosenberg et al. (2014), Lee et al. (2017), and Rosenberg et al. (2018) conducted a randomized controlled trial (RCT) and assessed whether the resilience outcome(s) improved after the intervention (Lee et al., 2017; Rosenberg et al., 2014, 2018). Moreira et al. (2015), Lee et al. (2020), and Zimmerman et al. (2021) compared resilience scores (as an outcome of resilience) of children with a (specific) chronic disease and a control group (Lee et al., 2020; Moreira et al., 2015; Zimmerman et al., 2021). Additionally, Kaewkong et al. (2020) examined whether demographics such as sex and age were associated with the resilience outcome (Kaewkong et al., 2020). The remaining 7 (13%) studies assessed resilience factors only. To illustrate, Whiteley et al. (2019) and Hood et al. (2018) conducted RCTs with the aim to assess whether their intervention improved certain resilience factors, such as treatment motivation, of the participants (Hood et al., 2018; Whiteley et al., 2019).

### Resilience Outcomes

A myriad of resilience outcomes has been assessed in the included studies (see Fig. 2). For each instrument, we examined which topic(s) was/were being measured by searching the corresponding or background articles (for references see Supplement 3). This examination resulted in three categories: *personal traits*, *psychosocial functioning*, and *disease-related outcomes*. In total, 34 instruments were used: 9 (26.5%) instruments assessed resilience by means of outcomes related to personal traits (Achenbach & Rescorla, 2001; Bahryni et al., 2016; Campbell-Sills & Stein, 2007; Dias et al., 2014; Haase et al., 2014; Kim & Yoo, 2010; Merell, 2011; Prince-Embury, 2007; Wagnild & Young, 1993), 9 (26.5%) instruments appraised resilience by outcomes of psychosocial functioning (Achenbach, 1991; Bouma et al., 1995; Goodman, 1997; Laurent et al., 1999; Nabors et al., 2021; Pynoos et al., 1998; Roberts et al., 1993; Thompson & Gray, 1995; Varni et al., 1999a, b), 13 (38%) instruments consisted of disease-related outcomes (Currier et al., 2009; Fenton et al., 2015; Huston et al., 2016; Jaser & White, 2011; Markowitz et al., 2010; Phipps et al., 2007; Schwartz & Drotar, 2009; Varni et al., 1999a, b; Welch et al., 1997; Wood et al., 2014; Yang et al., 2012), 2 (6%) instruments measured both personal traits and psychosocial functioning (Beck et al., 2005; Reynolds & Kamphaus, 2004), and 1 (3%) instrument assessed both personal traits and disease-related outcomes (Kim & Yoo, 2010). Examples of personal traits are self-efficacy measured by the General Self-Efficacy Questionnaire (GFE-10) (Bahryni et al., 2016), responsibility measured by the Social-Emotional Assets and Resilience Scales (SEARS) (Merell, 2011), and affect measured by the Positive and Negative Affect Scale for Children (PANAS-c) (Laurent et al., 1999). Examples of psychosocial functioning are quality of life measured by the Pediatric Quality of Life Inventory (PedsQL) (Varni et al., 1999a, b), (absence of) internalizing symptoms measured by the Center for Epidemiologic Studies Depression Scale (CESDS) (Bouma et al., 1995), and (absence of) post-traumatic stress disorder symptoms measured by the UCLA PTSD Reaction Index for DSM-IV (PTSDI) (Pynoos et al., 1998). Examples of disease-related outcomes are disease-related quality of life measured by the disease-specific modules of the PedsQL (Varni et al., 1999a, b) and benefit finding in illness measured by the Benefit Finding Scale for Children (BFSC) (Phipps et al., 2007).

In total, 13 (24% of all included) studies used a resilience instrument to measure positive adaptation to stress (resilience outcome) by calculating a total resilience score per participant rather than assessing each topic separately. Examples of the scales used to derive total resilience scores are the Connor-Davidson Resiliency Questionnaire (CD-RISC) (Rosenberg et al., 2014, 2018; Verma & Rohan, 2020; Zimmerman et al., 2021), the Wagnild and Young Resilience Scale (RS) (Gmuca et al., 2021; Lee et al., 2019; Lee et al., 2014, 2017, 2020; Moreira et al., 2015; Wu et al., 2018), the Healthy Kids Resilience Assessment Module (Santos et al., 2016), and the Family Resilience Assessment Scale (FRAS-C) (Cui et al., 2022).

When focusing on the resilience outcomes regarding psychosocial functioning, both positive and negative outcomes were assessed; concerning the latter, participants were considered resilient when they reported an absence of mental health problems.

All included instruments were questionnaires, except for the HbA1c which is a blood test, used to assess metabolic control (Jaser & White, 2011).

### Resilience Factors

We categorized the resilience factors into *internal factors*, *disease-related factors*, and *external factors*. Internal factors comprised cognitive, emotional, and social competence factors. External factors included caregiver factors, peer factors, and contextual factors. Disease-related factors could be both internal and external factors (Fig. 3; Supplement 4).

In total, 66 different instruments were used to measure resilience factors. Overall, 33 (50%) instruments assessed internal factors, 15 (23%) instruments assessed disease-related factors, 11 (17%) instruments assessed external factors, and 7 (10%) instruments assessed a combination of internal, disease-related, or external factors (Supplement 4). Figure 3 shows which resilience factors are measured. With regard to internal factors, examples of cognitive, social, and emotional competence factors were measured, such as coping (Barger et al., 2017; Carver, 1997; Endler & Parker, 1999; Hood et al., 2018; Jalowiec, 1984; Sandler et al., 2000), social skills (Achenbach, 1991; Achenbach & Rescorla, 2001; Gartland et al., 2011; Liebenberg et al., 2012), and self-efficacy (Campbell-Sills & Stein, 2007; Connor & Davidson, 2003; Martins, 2005). Some examples of the disease-related factors were acceptance of the disease (Evers & Kraaimaat, 2009; McCracken et al., 2010), social support promoting adherence (Cutrona & Russel, 1987; Fisher et al., 2006), and disease-related coping (Connor-Smith et al., 2000; Gil et al., 1991; Wu et al., 2011). With regard to external resilience factors, caregiver factors focused, among others, on family cohesion and connectedness (Gartland et al., 2011; Karcher, 2005; Karcher & Sass, 2010; Lim et al., 1990; Olson, 1985); peer factors focused, among others, on peer relations (Haase et al., 2014; Hilliard et al., 2017; Liebenberg et al., 2012; Martins, 2005; Merell, 2011); and contextual factors, focused, among others, on spiritual, educational, and cultural environment (Liebenberg et al., 2012).

Out of all included studies, 10 (18%) employed a resilience instrument as a resilience factor by calculating a total resilience score per participant, as opposed to measuring individual domains separately. Examples of the scales used to derive total resilience scores as resilience factor are the resilience measurement instrument for children with chronic illness (Kim & Im, 2014), the Wagnild and Young Resilience Scale (RS) (Chung et al., 2021; Gmuca et al., 2021), the Connor-Davidson Resiliency Questionnaire (CD-RISC) (Bahryni et al., 2016; Verma & Rohan, 2020), Haase Adolescent Resilience in Illness Scale (HARIS) (Huang et al., 2018), 7Cs Tool (Borinsky et al., 2019), The Neil and Dias Resilience Scale (Lukacs et al., 2018), Diabetes Strengths and Resilience Measure for Adolescents (DSTAR) (Araia et al., 2020), and the Child and Youth Resilience Measure (CYRM-28) (Kully-Martens et al., 2021).

## Discussion

The aim of this scoping review was to provide an overview of definitions and instruments used to assess resilience in children with a chronic disease. In total, 55 articles were included. Resilience was often conceptualized as a dynamic concept that signals a positive adaptive response to stress or adversity. The included studies either assessed resilience by the outcomes of positive adaptation or by resilience factors. Most studies (65%), however, measured both resilience outcomes and resilience factors simultaneously and examined whether resilience factors were associated with a resilience outcome. We categorized the assessed resilience outcomes into three groups: personal traits, psychosocial functioning, and disease-related outcomes. Moreover, myriad of resilience factors were measured, which were grouped into internal resilience factors (cognitive, social, and emotional competence factors), disease-related factors, and external factors (caregiver factors, social factors, and contextual factors). Disease-related factors could be both internal and external factors.

Even though resilience has been a topic of interest in scientific research since the 1970s, the articles that met our inclusion criteria were relatively recent (2009–2022) (Zolkoski & Bullock, 2012). We did not add restriction in terms of publication date as exclusion criteria in the search. The relatively recent publication dates of the included articles suggest that resilience has recently gained more (scientific) attention in pediatric healthcare research.

Although we observed many commonalities between the used definitions, this review identified that one cannot simply compare “resilience” in one paper with “resilience” in other papers. Therefore, just as the new agreement on the concept of health, a global definition and agreement on terminology of resilience would be helpful in crossing the boundaries between medical research and other relevant disciplines to enhance our understanding of general, trans-diagnostic, and disease-specific aspects of resilience factors and outcomes. By differentiating between factors and outcomes, and between internal, disease-related, and external factors, this scoping review makes a first attempt to reach a consensus on the conceptualization of resilience. The resilience definition of Luthar et al. (2000) was cited most frequently by the articles included in this review: “Resilience refers to a dynamic process encompassing positive adaptation within the context of significant adversity” (Luthar et al., 2000). In our opinion, however, it would be helpful to employ a definition that also identifies (one) measurable outcome(s) of positive adaptation.

Various resilience outcomes have been measured by the included studies. Resilience outcomes reflected positive adaptation to stress, which was measured, for example, by pediatric QoL, child competence, and (lack of) mental health problems. Although resilience is defined as a positive adaptation to stress, many mental health instruments measured (the absence of) negative outcomes, such as emotional and behavioral problems (Kaewkong et al., 2020; Kim & Im, 2014; Sharp et al., 2018), anxiety and depressive symptoms (Rassart et al., 2016; Simon et al., 2009), loneliness (Rassart et al., 2016), or posttraumatic stress symptoms (Sharp et al., 2015). Children were identified as being resilient when they did not experience these symptoms or problems. Measuring a resilience outcome as the absence of negative outcomes introduces several challenges, leading one to question whether positive adaptation to a stressor should be measured as such. First, there are limitations to assessing binary outcomes. By examining continuous scores, we can advance our understanding of, for instance, clinical and sub-threshold problems. Both clinical and sub-threshold problems can be debilitating in the everyday life of children with a chronic disease. Therefore, focusing on absence versus presence of certain disorders is not an ideal resilience outcome. Secondly, we might be careful using the absence of negative outcomes because not having a disorder/symptom/problem does not necessarily imply that one has a good mental health. To illustrate, the definition of wellbeing is: life satisfaction, the presence of positive affect, and the absence of negative affect. The presence of positive affect does not mean that negative affect is absent or vice versa. Positive and negative affect are — although negatively correlated — partly independent dimensions of well-being (Diener, 1984; Rao et al., 2017; Smith et al., 2021; Weich et al., 2011). How could researchers measure resilience outcomes in a positive manner and which outcomes can be used to do so? In recent decades, the outlook on health has shifted from an approach merely focusing on (the absence of) physical health to a more dynamic and all-encompassing concept assessing various dimensions of well-being and psychosocial functioning: the ability to adapt and self-manage in the face of social, physical, and emotional challenges (Huber et al., 2011). In total, six dimensions have been described that influence adaption: bodily functions, mental functions and perceptions, spiritual dimension, quality of life, social and societal participation, and daily functioning (Huber et al., 2016). All dimensions, except bodily functions, might be used as positive resilience outcomes regarding psychosocial functioning and positive mental health. When measuring only improvement or adaptation of bodily functions, one focuses on physical resilience (Whitson et al., 2016). Although we selected articles focusing on psychological resilience, some of the included studies combined physical and psychological resilience (Jaser & White, 2011; Yi-Frazier et al., 2015) As an example, Jaser and White explored how the use of specific coping strategies impacts resilience (defined as quality of life, competence, and metabolic control) among adolescents with type 1 diabetes (Jaser & White, 2011). To do so, they incorporated both psychological and physical resilience outcomes, conceptualizing that physical and psychological resilience are intertwined.

Most of the internal and external resilience factors, measured in the current scoping review, have also been reported in other (systematic) reviews focusing on resilience factors associated with different adversities, such as childhood maltreatment, war, and poverty (Afifi & MacMillan, 2011; Fritz et al., 2018; Gartland et al., 2019). Noteworthy, in the current scoping review many disease-related factors were also identified as resilience factors. These factors might be new targets for psychosocial interventions to improve children’s positive adjustment to their chronic disease. Moreover, in previous literature, multiple levels of the environment are described (Bronfenbrenner, 1979): proximal levels, which include the child’s direct relationships, such as relationships with parents and friends (Afifi & MacMillan, 2011; Rutter, 1985; van Harmelen et al., 2017) and more distal levels such as characteristics of the neighborhood or culture of society (Bronfenbrenner, 1979). When focusing on the external factors measured by the studies in this review, most of these factors were related to proximal levels of the environment and included the child’s relationships with caregivers (caregiver factors) and with peers (peer factors). Although some factors seemed to be more distal, such as expectations at school or connectedness with the neighborhood (Karcher, 2005; McNeely et al., 2002) (contextual factors), all factors focused on the child’s perception of the school or neighborhood. This is the result of including only self-reported instruments in this review. Although the more distal factors, such as cultural norms or the effect of time on the adversity, are not easily measured with questionnaires, these elements could be very important for positive adaptation to a chronic disease. For instance, the organizational culture of hospitals may have an impact on the shared ways of thinking, feeling, and behaving of doctors, which might influence prevailing views on patient needs and therefore the openness of doctors to their patients’ input (Mannion & Davies, 2018). Huber showed that adult patients’ views on health are much broader than that of doctors: patients give equal importance to bodily functions as QoL, spirituality, and mental state, while doctors focus predominantly on bodily functions (Huber et al., 2016). When medical professionals are used to invite (pediatric) patients to express their feelings and experiences not only about their bodily functions, but also about other important aspects of their lives (e.g., friendships, and mental well-being), adaptation to their disease might be enhanced. Furthermore, positive adaptation to a chronic disease may change or develop over time, and therefore, researchers might consider when and over which time frame resilience should be measured. Disease severity and the frequency of relapses or exacerbations might be taken into consideration too, as these aspects of a chronic disease could play a role in the adaptation process. Finally, many leading resilience researchers not only acknowledge that internal factors and external factors facilitate resilience, but also emphasize the importance of interaction between the child and their environment (Ioannidis et al., 2020; Kalisch et al., 2017; Masten, 2015; Southwick et al., 2014).

Several strengths of our review deserve mentioning. First, we conducted a broad literature search across multiple electronic databases. This resulted in a diverse samples of articles, half of which were published in the last decade, representing many different chronic diseases in various countries across the world. Furthermore, this search offered a comprehensive overview of definitions and how resilience is measured: as an outcome of positive adaptation to a stressor, as resilience factor(s), or both. Moreover, our scoping review’s first attempt to reach a consensus on the conceptualization of resilience is by categorizing resilience measurements by between factors and outcomes. Some limitations need to be mentioned. Our inclusion criteria involved resilience, (chronic) disease, and children — in combination with the corresponding MESH terms. We did not add terms that describe the functions of resilience such as “buffering” or “adaptation” to the search string. This might have resulted in missing potentially relevant articles. Additionally, some articles identified by the used search terms described researched resilience but lacked information on how resilience was operationalized. These articles were also not included in this review. We hope that our review will stimulate future investigations that include research describing the functions of resilience. Furthermore, as we chose to only include self-report measurements, we implicitly excluded studies with children younger than 6 years old, as well as the parental perspective on resilience. Research indicates that children are able to report on their health-related quality of life from the age of 5 years (Varni et al., 2007), and most resilience questionnaires are deemed appropriate for self-reporting from the age of 8 years (King et al., 2021; Vannest et al., 2021) (see Supplement 2, indicating the age of the populations studied).

It was beyond the scope of this review to evaluate whether the resilience factors were actually (significantly) associated with the resilience outcome. Therefore, we are unable to conclude whether these factors contribute to positive adaptation to stress. Notwithstanding, this provides several interesting avenues for further research. The first are the biological mechanisms underpinning resilience factors. In this systemic review, we did not report on the working mechanism of a resilience factor, and therefore we were unable to answer why the identified resilience factors facilitated positive adaptation to disease-related challenges in children with a chronic disease. Multiple mechanisms explaining resilience in the face of childhood adversity have been described, involving biomedical processes at the genetic, inflammation and brain level and involving processes in external levels (Ioannidis et al., 2018; Kalisch et al., 2017, 2019). Future research in pediatric healthcare could examine if these mechanisms also explain positive adaptation of disease-related challenges. Furthermore, it is acknowledged that positive adaptation to stress is not facilitated by one resilience factor only, but rather it is an interplay between multiple factors. Therefore, an insight into how these underlying mechanisms across internal and external levels interact would also enhance our understanding of resilience in children with a chronic disease. Furthermore, it should be further elucidated whether underlying mechanisms differ across different diseases. A second aspect is disease-specific associations. As chronic diseases vary in terms of predictability, treatment regimen, side effects, life-expectancy, disability, and impact on daily functioning, it is conceivable that resilience factors have a different contribution to positive adaptation in different disease-related challenges. For instance, self-efficacy of treatment management and self-esteem were identified in this review as internal factors that contribute to resilience. However it is possible that the degree to which these factors facilitate positive adaptation differs per disease. To illustrate: when looking at resilience in pediatric cancer patients, more emphasis may be on decreased self-esteem due to changes in physical appearance, whereas self-efficacy of treatment management might be less challenged, as cancer treatment is typically administered in hospital settings. To gain more insight into which factors are related to positive adaptation to specific disease-related challenges, it might be useful to use instruments that include questions on disease-specific challenges. Lastly, a third aspect revolves around disease severity. Several articles acknowledged that having a chronic disease or experiencing symptoms of the disease is a stressor; however, the severity of the disease was not often taken into account in the analyses. Van Harmelen et al. showed that taking the severity of the stressor into account is of importance when researching resilient functioning (van Harmelen et al., 2017, 2020). They quantified resilient functioning as the degree to which the child shows better or worse psychosocial functioning than expected, given their experienced adversity. The researchers define psychosocial functioning as an outcome of positive adaptation to adversity. They identified that the resilience factors *parent support* and *friendships* were significantly more present in children who functioned better than expected given their experienced stress, than in children who functioned worse than expected given their experienced stress (van Harmelen et al., 2017).

In short, our scoping review on resilience in children with a chronic disease provides insight into the variety of definitions and the multidimensionality of resilience outcomes and resilience factors in pediatric healthcare research. Research may profit from a shared definition that facilitates comparability and enhances our understanding of resilience in the pediatric healthcare field. Moreover, future research might focus on which resilience factors are related to positive adaptation in specific disease-related challenges, which underlying mechanisms are responsible for this positive adaptation, and how these underlying mechanisms interact with one another. These insights could be used to develop new psychosocial interventions to stimulate resilience of children with a chronic disease.

## Supplementary Information

Below is the link to the electronic supplementary material.

Supplementary file1 (DOCX 16.5 KB)

Supplementary file2 (DOCX 488 KB)

Supplementary file3 (DOCX 111 KB)

Supplementary file4 (DOCX 214 KB)

Supplementary file5 (DOCX 108 KB)

## Diseases/therapy

ART: Antiretroviral treatment
BMT: Bone marrow transplantation
CHD: Congenital heart disease
CKD: Chronic kidney disease
DMD: Duchenne muscular dystrophy
HIV: Human deficiency virus
HSCT: Hematopoietic stem cell transplant
IBD: Inflammatory bowel diseases
MS: Multiple sclerosis
SCD: Sickle cell disease
T1D: Type 1 diabetes

## Instruments

ACT: Asthma Control Test
AFQ-Y: Avoidance and Fusion Questionnaire for Youth
ARC: Adolescent Resilience Questionnaire
ASRI: Adolescent Self-Regulatory Inventory
ATQ: Automatic Thoughts Questionnaire
BASC: Behavior Assessment System for Children
BASC-2 SRP: Behavior Assessment System for Children 2nd edition Self-Report of Personality
BBSC: Benefit Finding/Burden Scale for Children
BFSC: Benefit Finding Scale for Children
BSCI-Y: Chinese Beck Self-Concept Inventory
CASQ-R: Children’s Attributional Style Questionnaire-Revised
CATIS: Child Attitude Toward Illness Scale
(K-) CBCL: Child Behavior Checklist
CD-RISC (-10/-25): Conner-Davidson Resiliency Questionnaire
CDRS: Contour Drawing Rating Scale
CEQ: Coping Efficacy Questionnaire
CESDS: Center for Epidemiologic Studies Depression Scale
CHS: Children’s Hope Scale
CICRS: Chronic Illness Children’s Resilience Scale
CISS: Coping Inventory for Stressful Situations
brief COPE: Coping orientation to problems experienced
CPAQ-A: Dutch Chronic Pain Acceptance Questionnaire — Adolescent Version
CSES: Child Self-Efficacy Scale
CSQ: Coping Strategies Questionnaire for Sickle Cell Disease
CYRM-28: Child and Youth Resilience Measures-28
DEPS-R: Diabetes Eating Problem Survey-Revised
DSMP: Diabetes Self-Management Profile
DSTAR-Teen: Diabetes strengths and resilience measure for adolescents
Diseases: COVID-19: Coronavirus disease 2019
EAC: Emotional Approach & Coping Scale
EFI: Eco-cultural family interview
FACES III: Family Adaptability and Cohesion Scale
FAMSS: Family Asthma Management System Scale
FRAS-C: Family Resilience Assessment Scale
GSE-10: General Self-efficacy Questionnaire
HARIS: Haase Adolescent Resilience in Illness Scale
HbA1c: Hemoglobin A1c
HMAC: Hemingway measure of adolescent connectedness
ICQ: Illness Cognition Questionnaire
KBIT-2: Kaufman Brief Intelligence Test: Second Edition
K6: Kessler-6 Psychological Distress Scale
LKQCHD: Leuven Knowledge Questionnaire for Congenital Heart Disease
LOT: Life Orientation Test
PACE-Adolescent: Patient-based assessment and counseling for physical activity and nutrition-adolescent
PAID: Problem Areas in Diabetes Scale
PANAS-C: Positive and Negative Affect Scale for Children
PCCS: Pediatric Cancer Coping Scale
PedsQL: Pediatric Quality of Life Inventory
PPVT-III: Peabody Picture Vocabulary Test — Third Edition
PTSDI: University of California, Los Angeles Post-Traumatic Stress Disorder Reaction Index for Diagnostic and Statistical Manual of Mental Disorders
PSI-SF: Parenting Stress Index — Short Form
RS: The Wagnild and Young Resilience Scale
RSCA: Resilience Scale for Children and Adolescents
RSQ: Responses to Stress Questionnaire
SCS: School Connectedness Scale
SDQ: Strengths and Difficulties Questionnaire
SEARS: Social-Emotional Assets and Resilience Scales
SED: Self-efficacy for diabetes
SPSI-R:S: Social Problem-Solving Inventory-Revised short form
SSS: School Support Scale
SSSS: Scale of Satisfaction with Social Support
STARx: Self-Management and Transition to Adulthood with Rx = Treatment
TRAQ: Transition Readiness Assessment Questionnaire
YSR: Youth Self-Report

## Other

RCT: Randomized controlled trial
QoL: Quality of life
MESH: Medical Subject Headings
PRISMA: Preferred Reporting Items for Systematic Reviews and Metaanalyses
