# Supplementary material for: Defining and Measuring Resilience in Children with a Chronic Disease: a Scoping Review
Source: Advers Resil Sci. 2023 Apr 10;4(2):105–23. doi: 10.1007/s42844-023-00092-2 (PMC10088629; doi:10.1007/s42844-023-00092-2)
Supplement: Supplementary file 1 — Supplementary file1 (DOCX 16.5 KB) [file 42844_2023_92_MOESM1_ESM.docx]

**Supplement 1: Search strings (search performed on December 9, 2022)**

PubMed (items = 1531):

("Chronic Disease"[MeSH] OR “disease”[MeSH] OR "disease*"[tiab] OR "illness*"[tiab] OR "condition*"[tiab] OR "sickness*"[tiab] OR "disorder*"[tiab]) AND ("child"[MeSH] OR "adolescent"[MeSH] OR "child*"[tiab] OR "adolescen*"[tiab] OR "minor*"[tiab] OR "juvenile*"[tiab] OR "kid*"[tiab] OR "young*"[tiab] OR pediatrics[MESH] OR pediatri* [tiab] OR paediatri*[tiab] OR boy[tiab]  OR boys[tiab] OR girl[tiab]  OR girls[tiab] OR schoolchild*[tiab]  OR school child*[tiab] ] OR  youth[tiab] OR youths[tiab] OR teen[tiab] OR teens[tiab] OR  teenager[tiab]) AND (“Resilience, Psychological”[MeSH] OR "resilien*"[tiab])

Embase (items = 1388):

(‘chronic disease’/exp OR 'diseases'/exp OR disease*.ti,ab,kw OR illness*.ti,ab,kw OR condition*.ti,ab,kw  OR sickness*.ti,ab,kw OR disorder*.ti,ab,kw) AND ('child'/exp OR 'adolescent'/exp OR adolescen*.ti,ab,kw OR minor*.ti,ab,kw OR juvenile*.ti,ab,kw OR kid*.ti,ab,kw OR young*.ti,ab,kw OR pediatri*.ti,ab,kw OR paediatri*.ti,ab,kw OR boy.ti,ab,kw OR boys.ti,ab,kw OR girl.ti,ab,kw OR girls.ti,ab,kw OR schoolchild*.ti,ab,kw OR child*.ti,ab,kw OR youth.ti,ab,kw OR youths.ti,ab,kw OR teen.ti,ab,kw OR teens.ti,ab,kw OR teenager.ti,ab,kw) AND (‘psychological resilience’/exp OR resilien*.ti,ab,kw)

PsycINFO (items = 4397):

(exp Chronic Illness/ or disease.mp. or (disease* or illness* or condition* or sickness* or disorder*).ti,ab.) and (child.mp. or adolescent.mp. or (child* or adolescen* or minor* or juvenile* or kid* or young* or pediatric* or paediatri* or boy or boys or girl or girls or schoolchild or youth or youths or teen or teens or teenager).ti,ab.) and (exp "Resilience (Psychological)"/ or resilien*.ti,ab.)

Cochrane (items = 1450):

((MeSH descriptor: [Chronic Disease] explode all trees) OR (MeSH descriptor: [Disease] explode all trees) OR ((disease*):ti,ab,kw OR (illness*):ti,ab,kw OR (condition*):ti,ab,kw OR (sickness*):ti,ab,kw OR (disorder*):ti,ab,kw)) AND ((MeSH descriptor: [Child] explode all trees) OR (MeSH descriptor: [Adolescent] explode all trees) OR ((child*):ti,ab,kw OR (adolescen*):ti,ab,kw OR (minor*):ti,ab,kw OR (young*):ti,ab,kw OR (kid*):ti,ab,kw)) AND ((MeSH descriptor: [Resilience, Psychological] explode all trees) OR ((resilien*):ti,ab,kw))
