# Supplementary material for: Defining and Measuring Resilience in Children with a Chronic Disease: a Scoping Review
Source: Advers Resil Sci. 2023 Apr 10;4(2):105–23. doi: 10.1007/s42844-023-00092-2 (PMC10088629; doi:10.1007/s42844-023-00092-2)
Supplement: Supplementary file 2 — Supplementary file2 (DOCX 488 KB) [file 42844_2023_92_MOESM2_ESM.docx]

| **First Author (year),**  ***Disease*** | **Study design** | **n** | **Age range** or **mean**  ± **SD (years)** | **Country** | **Definition of resilience** | **Originally defined by** | **Assessment of resilience** | |
| --- | --- | --- | --- | --- | --- | --- | --- | --- |
|  |  |  |  |  |  |  | **●**  ***Resilience outcome*** | **●**  ***Resilience***  ***factor(s)*** |
| **Atopic diseases** | | | | | | | | |
| (LeBovidge et al., 2009)  *Food allergy* | Cross-sectional | 141 | 8-17 | USA | No definition | NA | **●** Behavior Assessment System for Children Self-Report of Personality (BASC-2 SRP)  **●** Child Attitude Toward Illness Scale (CATIS) | |
| (Im & Kim, 2012)  *Atopic dermatitis* | Cross-sectional | 102 | 7-15 | South Korea | “(…) individuals may overcome difficulties and adapt better using their positive capacities and available resources even under the risky and challenging situation, such as diagnosing a chronic disease interfering the child and family’s life.” | (Davis, 2004) | **●** Resilience measurement instrument for children with chronic illness  **●** Childrearing Behavior Questionnaire | |
| (Kim & Im, 2014)  *Atopic dermatitis* | Cross-sectional | 120 | 7-13 | South Korea | “(…) the ability to function with healthy responses despite the presence of significant stress and adversity” | (A. S. Masten & O’Connor, 1988) | **●** Child Behavior Checklist (K-CBCL)  **●** Resilience measurement instrument for children with chronic illness | |
| (Nabors et al., 2021)  *Asthma* | Cross-sectional | 2383 | 12-17 | USA | “Family resilience involves adjustment or rebounding in the face of stress. Family resilience can involve problem-solving, interacting positively with each other, and at the same time be influenced by a multitude of environmental stressors experienced by family members.” | (Buehler, 2020), (Walsh, 2016), (Traub & Boynton-Jarett, 2017) | **●** Family Resilience: NSCH-subscale ‘family flourishing’ | |
| **Auto-immune disorders** | | | | | | | | |
| (Carlsen et al., 2017)  *Inflammatory Bowel Diseases* | Cross-sectional | 87 | 16-23 | USA | “One's ability to bounce back from obstacles.” | (Iacoviello & Charney, 2014) | **●** Transition Readiness Assessment Questionnaire (TRAQ)  **●** Connor-Davidson resiliency questionnaire (CD-RISC-10) | |
| (Beeckman et al., 2019)  *Juvenile idiopathic arthritis* | Cross-sectional | 59 | 13.8 ± 2.67 | Belgium | “(…) resilience factors is crucial if we are to understand how, why, and in which contexts some individuals function well despite pain whereas others do not.” | (Goubert & Trompetter, 2018), (Cousins et al., 2015), (Sturgeon & Zautra, 2010) | **●** Pediatric Quality of Life Inventory (PedsQL)  **●** Positive and Negative Affect Scale for Children (PANAS-C)  **●** Psychological flexibility: Avoidance and Fusion Questionnaire for Youth (AFQ-Y)  **●** Dutch Chronic Pain Acceptance Questionnaire– Adolescent version (CPAQ-A) | |
| (Gmuca et al., 2021)  *Juvenile fibromyalgia syndrome* | Cross-sectional | 31 | 12-17 | USA | “Resilience serves as an important protective factor  against psychological distress. Resilience can be  defined as a dynamic process of positive adaptation or continued development in the context of adversity (e.g. chronic pain).” | (A. S. Masten, 2001), (Luthar et al., 2000) | **●**14-item Resilience Scale (RS-14) ﻿ | |
| (Tang et al., 2022)  *Henoch Schonlein purpura nephritis* | Longitudinal | 60 | 9.15 ± 1.59 | China | No definition | NA | **●** Chronic Illness Children’s Resilience Scale (CICRS) | |
| **Malignancies** | | | | | | | | |
| (Kim & Yoo, 2010)  *Cancer (nfs)* | Cross-sectional | 74 | 10-15 | South Korea | “The concept of resilience came from the paradigm that individuals could overcome difficulties and adapt better by utilising strengths and abilities that they already have.” | NR | **●** Resilience measurement instrument for children with chronic illness  **●** Family Adaptability and Cohesion Scale (FACES III)  **●** Relationship with friends: Personal Relationship Measurement  **●** Relationship with teachers: School Adjustment Test | |
| (L. M. Wu et al., 2013)  *Cancer (nfs)* | Cross-sectional | 131 | 11-19 | Taiwan | “(…) the ability to recover from or overcome negative events in terms of mental health, functional capacity, and social competence” | (Hunter & Chandler, 1999) | **●** Haase Adolescent Resilience in Illness Scale (HARIS)  **●** Pediatric Cancer Coping Scale (PCCS) | |
| (Robb et al., 2014)  *Hematopoietic Stem Cell Transplant* | RCT | 113 | 11-24 | USA | “The process of identifying or developing resources and strengths to flexibly manage stressors to gain a positive outcome, a sense of confidence, mastery, and self-esteem.” | (J. E. Haase, 2004) | ● Haase Adolescent Resilience in Illness Scale (HARIS)  ● McCorkcle Symptom Distress Scale  ● Mishel Uncertainty in Illness Scale  ● Jalowiec Coping Scale-Revised  ● Reed Spiritual Perspective Scale  ● Perceived social support from healthcare providers, friends and family: Perceived Social Support  ● Family adaptability/Cohesion Scale II  ● Parent-Adolescent Communication Scale  ● Family Strengths Scale  ● Hope-Derived Meaning: Herth Hope Index | |
| (K. Sharp et al., 2015)  *Cancer (nfs)* | Cross-sectional case-control | 254 | 8-19 | USA | No definition | NA | **●** UCLA PTSD Reaction Index for DSM-IV (PTSDI)  **●** Benefit Finding/Burden Scale for Children (BBSC)  **●** Hemingway Measure of Adolescent Connectedness (HMAC) | |
| (Bahryni et al., 2016)  *Cancer (nfs)* | Cross-sectional | 120 | NR | Iran | “(…): resiliency is defined as human adaptability in confrontation with overwhelming disasters or pressures, overcoming and even get strengthen by them. It can be said that resiliency is an individual’s ability to make bio-psycho balance in dangerous conditions.” | (Diener et al., 2003), (Connor & Davidson, 2003) | **●** General self-efficacy questionnaire (GSE-10)  **●** Connor-Davidson resiliency questionnaire (CD- RISC-25) | |
| (Schwartz & Brumley, 2017)  *Cancer (nfs)* | Cross-sectional case-control | 199 | 13-19 | USA | No definition | NA | **●** Health-related Hindrance Inventor  **●** Brief Coping Orientation to Problems Experienced (COPE)¶  **●** Cowen Self-Efficacy Scale  **●** Childen’s Hope Scale (CHS)  **●** Family Assessment Device  **●** Perceived Social Support Scale | |
| (Rosenberg et al., 2018)  *Cancer (nfs)* | RCT | 92 | 12-25 | USA | “The process of harnessing resources to sustain physical and emotional well-being in the face of significant stress.” | (Bonanno et al., 2011) | **●** Coping Orientation to Problems Experienced (CD-RISC-10) | |
| (Willard et al., 2018)  *Brain tumor* | Prospective study | 53 | 13.11 ± 2.31 | USA | No definition | NA | **●** Social-Emotional Assets and Resilience Scales (SEARS¶)  **●** Hemingway Measure of Adolescent Connectedness (HMAC) | |
| (W. Wu et al., 2018)  *Cancer (nfs)* | Cross-sectional | 40 | 10-18 | Taiwan | “(…) the capacity of individuals to successfully maintain their psychosocial well-being in the face of adversity, is a positive health concept that may be used to improve psychosocial well-being and quality of life in adolescents with cancer.” | (Stewart & Yuen, 2011), (J. E. Haase et al., 2014) | **●** The Wagnild and Young Resilience Scale (RS)  **●** Beck Self- Concept Inventory (BSCI-Y) | |
| (J. Y. Lee et al., 2019)  *Leukemia* | Cross-sectional | 72 | 13-20 | South Korea | “The ability to adapt to situations and environments through self-regulation.” | (Gheshlagh et al., 2016) | **●** The Wagnild and Young Resilience Scale (RS)  **●** Illness Cognition Questionnaire (ICQ) | |
| (Lau et al., 2020)  *non-central nervous system cancer* | Longitudinal | 14 | 14-25 | USA | “(…) a process of harnessing resources needed to sustain individual well-being.” | (J. Haase et al., 1999),(Southwick et al., 2014), (Rosenberg et al., 2014) | **●** Connor-Davidson resiliency questionnaire (CD-RISC-10¶)  **●** Parental distress: Kessler-6 psychological distress scale (K6) | |
| (Chung et al., 2021)  *Leukemia, lymphoma, brain tumor, osteosarcoma, kidney tumor* | Cross-sectional | 138 | 7-14 | China | ﻿“Resilience is defined as an individual's strength and ability to moderate the negative effects of stress, promote adaptation, and maintain mental well‐being in the face of adversity.” | (Wagnild & Young, 1993), ﻿ (Davydov et al., 2010) | **●** Pediatric Quality‐of‐Life Inventory Cancer Module version 3.0 (PedsQL Cancer Module)  **●** 14-item Resilience Scale (RS-14) ﻿ | |
| **Congenital Heart Disease** | | | | | | | | |
| (S. Lee et al., 2014) | Cross-sectional | 103 | 14-20 | South Korea | “(…) a dynamic process encompassing positive adaptation within the context of significant adversity; it is the ability to sustain adaptive functioning and positive growth and development in the face of significant stress.” | (Luthar et al., 2000),(Compas, 2006),(Mandleco & Peery, 2000) | **●** The Wagnild and Young Resilience Scale (RS)  **●** Coping Inventory for Stressful Situations (CISS) | |
| (Rassart et al., 2016) | Prospective study | 366 | 15-20 | Belgium | “Ego-resiliency refers to the tendency to respond flexibly to environmental demands.” | (Block & Block, 1980) | **●** Depression: Center for Epidemiologic Studies Depression Scale (CESDS)  **●** UCLA loneliness scale  **●** Pediatric Quality of Life Inventory (PedsQL)  **●** Cardiac module of the Pediatric Quality of Life Inventory (PedsQL)  **●** Personality Typology: Quick Big Five | |
| (S. Lee et al., 2017) | Controlled trial | 60 | 14-22 | South Korea | “(…) a dynamic process that encompasses positive adaptations to critical adversity, which is essentially the ability to maintain a pattern of positive responses and development in the face of crisis.”  “Resilience is associated with five psychosocial factors: (1) positive emotions (including optimism and humor), (2) cognitive flexibility (including positive explanatory style, positive appraisal and acceptance), (3) meaning (including religion, spirituality and altruism), (4) social support (including role models) and (5) an active coping style (including exercise and training).” | (Luthar et al., 2000), (Compas, 2006), (Southwick et al., 2005) | **●** The Wagnild and Young Resilience Scale (RS) | |
| (Huang et al., 2018) | Cross-sectional | 320 | 12-18 | Taiwan | “(…) the ability of an individual to successfully manage his or her life, and to successfully adapt to change and stressful events in healthy and constructive ways.” | (Dent, 2008) | **●** Health promoting behavior: Leuven Knowledge Questionnaire for Congenital Heart Disease (LKQCHD)  **●** Haase Adolescent Resilience in Illness Scale (HARIS) | |
| **Human Immunodeficiency Virus /** **Acquired Immune Deficiency Syndrome** | | | | | | | | |
| (C. Sharp et al., 2018) | Cross-sectional | 750 | 7-11 | USA | No definition | NA | **●** Behavioral and emotional problems: Strength and Difficulties Questionnaire (SDQ)  **●** School Connectedness Scale (SCS)  **●** Scale of Satisfaction with Social Support (SSS) | |
| (Whiteley et al., 2019) | RCT | 61 | 14-26 | USA | “Resilience, broadly defined as the capacity and skills to confront life challenges, can be a target for prevention and intervention programs for youth. Interventions can focus on resilience factors that can be measured and improved, such as self-efficacy, motivation, and support seeking.” | (Furniss et al., 2014), (Johnson et al., 2003), among others | ● Self-efficacy for ART use  ● ART motivation  ● Social support | |
| **Nonmalignant hematological diseases** | | | | | | | | |
| (Simon et al., 2009)  *Sickle Cell Disease* | Cross-sectional | 44 | 12-18 | USA | “ (…) resilience factors such as cognitive appraisals (i.e., hope and coping strategies) and intrapersonal characteristics (i.e., self-esteem and sense of inadequacy). The interplay among all these factors is thought to account for the variation in adaptation among adolescents with a chronic illness.” | (Wallander & Varni, 1998) | **●** Internalizing symptoms: Behavior Assessment System for Children (BASC)  **●** Children’s Attributional Style Questionnaire-Revised (CASQ-R)  **●** Children’s Hope Scale (CHS)  **●** Coping Strategies Questionnaire for Sickle Cell Disease (CSQ) | |
| (Kaewkong et al., 2020)  *Thalassemia* | Cross-sectional | 120 | 10-18 | Thailand | “(…) a dynamic ability to adapt successfully in the face of adversity, trauma, or significant threat.” | (Chmitorz et al., 2018), (A. S. Masten, 2014) | **●** Behavioral and emotional problems: Strengths and Difficulties Questionnaire (SDQ) | |
| (Wright et al., 2021)  *Sickle cell disease* | Cross-sectional | 93 | 12-18 | USA | “The process of resilience—an  individual’s ability to respond effectively to risk or adversity- (…).” | (A. S. Masten, 2001) | **●** Pediatric Quality of Life Inventory (PedsQL)  **●** Child and Adolescent Mindfulness Measure (CAMM)  **●** Chronic Pain Acceptance Questionnaire (CPAQA) | |
| (Parviniannasab et al., 2022)  *Hemophilia* | Cross-sectional | 372 | 11-21 | Iran | “(…) ﻿using one’s strength and capacity to adapt to stressful situations. Resilience is the ability and a personality characteristic which helps patients maintain their mental health when facing hardship and physical illnesses.” | (Connor & Davidson, 2003), (J. Haase et al., 1999) | **●** Connor-Davidson resiliency questionnaire (CD-RISC-10) | |
| **Nonmalignant neurological diseases** | | | | | | | | |
| (Fee & Hinton, 2011)  *Duchenne Muscular Dystrophy* | Retrospective | 165 | 6-14 | USA | “(…) ‘a dynamic process encompassing positive adaptation within the context of significant adversity’. Resilience is ‘the ability to maintain a stable equilibrium’ (…) It is not a particular personality trait but a process by which positive adaptation occurs despite adversity.” | (Werner, 1989) | **●** General psychosocial adjustment: Child Behavior Checklist (CBCL )  **●** IQ: Peabody Picture Vocabulary Test—Third Edition (PPVT-III)  **●** Social support: Child Behavior Checklist (CBCL)-social scale | |
| (Rainone et al., 2017)  *Multiple Sclerosis* | Cross-sectional | 53 | 14-24 | Italy | “A process through which the impact of a critical event is mitigated by the ability to overcome it by using and negotiating resources.” | NR | **●** Pediatric Quality of Life Inventory (PedsQL)  **●** Ego-Resiliency Scale  **●** Child and Youth Resilience Measures-28 (CYRM-28) | |
| (Arruda et al., 2021)  *Primary headaches* | Cross-sectional | 339 | 10-18 | Brazil | ﻿“Childhood resilience is a dynamic developmental process that reflects positive adaptation despite significant life adversities.” | (Luthar et al., 2000) | **●**﻿ Resilience Scale for Children and Adolescents (RSCA) | |
| (Zimmerman et al., 2021)  *Hydrocephalus* | Cross-sectional | 40 | 7-21 | USA | “(…) the process by which individuals use available resources to maintain well-being in the face of stress.” | (Bonanno et al., 2011) | **●** Connor-Davidson resiliency questionnaire (CD-RISC-10) | |
| (Cui et al., 2022)  *Epilepsy* | Cross-sectional | 1238 | 12-18 | China | “Resilience refers to the family's ability to withstand stressful experiences and rebound from them by creating new, healthy ways of functioning.” | (Walsh, 2003) | **●** Family Resilience Assessment scale (FRAS-C) | |
| **Obesity** | | | | | | | | |
| (Borinsky et al., 2019) | Cross-sectional | 85 | 13-21 | USA | “(…) the protective factors that dynamically allow one to have a good outcome, over-coming stress and adversity, while sustaining normal psychological and physical functioning.” | (G. Wu et al., 2013),(A. S. Masten, 2001) | **●** Body size dissatisfaction: Contour Drawing Rating Scale (CDRS)  **●** Resilience: 7Cs tool | |
| (Li et al., 2022)  *Metabolically healthy obesity (MHO)*  *Metabolically unhealthy obesity (MUO)* | Cross-sectional | 39 | MHO:  15.7 ±0.5  MUO:  15.3±0.4 | Canada | “A positive psychological adjustment in the face of adversity which is associated with improved health outcomes in patients with chronic conditions.” | (Wu et al., 2016) | **●** Adolescent Resilience Questionnaire (ARC) | |
| **Otolaryngology problems** | | | | | | | | |
| (Ruff et al., 2016)  *Orofacial anomalies* | Longitudinal study | 1196 | 7.5-18.5 | USA | “(…) an important factor among individuals facing potential adversity, and emotional and social functioning are critical for future development.” | NR | **●** Psychosocial functioning: Beck Youth Inventory for Emotional and Social Impairment (2nd edition)  **●** Resilience Scale for Children and Adolescents (RSCA) | |
| (Adibsereshki et al., 2021)  *Hearing loss* | Experimental | 122 | 12-15 | Iran | ﻿“Resilience refers to a dynamic process wherein individuals show positive adaptation despite experiences of significant adversity.” | (Luthar et al., 2000) | **●** ﻿The Adolescent Self-Regulatory Inventory (ASRI) | |
| **Type 1 Diabetes/ Type 2 Diabetes** | | | | | | | | |
| (Jaser & White, 2011)  *Type 1 Diabetes* | Cross-sectional | 30 | 10-16 | USA | “(…) factors that promote successful adaptation to type 1 diabetes. These positive outcomes in the context of a potentially adverse environment may be considered indicators of resilience.” | (Luthar et al., 2000), (Masten, 2007) | **●** Child Competence: Youth Self Report (YSR)  **●** Pediatric Quality of Life Inventory (PedsQL)  **●** Metabolic control: HbA1c¥  **●** Responses to Stress Questionnaire (RSQ) | |
| (Yi-Frazier et al., 2015)  *Type 1 Diabetes* | Pilot study | 50 | 13-18 | USA | “(…) is a construct describing an individual’s capacity to maintain psychological and/or physical well-being in the face of stress.”  “(…) personal resilience embodies combined personal resources including self-esteem, optimism, and self-efficacy.” | (Rew & Horner, 2003),  (Connor & Davidson, 2003)^,^  (Yi et al., 2008) | **●** Problem Areas in Diabetes Scale (PAID)  **●** Diabetes Self-Management Profile (DSMP)  **●** Pediatric Quality of Life Inventory (PedsQL)  **●** Metabolic control: HbA1c  **●** Optimism: Life Orientation Test  **●** Rosenberg Self-Esteem Scale  **●** Self-efficacy for diabetes (SED) | |
| (Huston et al., 2016)  *Type 1 Diabetes* | Cross-sectional | 243 | 11-16 | USA | No definition | NA | **●** Benefit Finding Scale for Children (BFSC)  **●** Diabetes acceptance, fitting in, and comfort in adjusting for diabetes in public*  **●** Emotion processing and regulation: Emotional Approach & Coping Scale (EAC) | |
| (Hood et al., 2018)  *Type 1 Diabetes* | RCT | 264 | 14-18 | USA | “Resilience consists of the following four key constructs: a sense of hopefulness, an optimistic explanatory style, effective coping strategies, and positive problem-solving skills.” | (Gillham et al., 2006) | **●** Resilience Scale for Children and Adolescents (RSCA)  **●** Automatic Thoughts Questionnaire (ATQ)  **●** Coping Efficacy Questionnaire (CEQ)  **●** Diabetes Strengths and Resilience measure for adolescents (DSTAR-Teen)  **●** Social Problem-Solving Inventory-Revised short form (SPSI-R:S) | |
| (Lukacs et al., 2018)  *Type 1 Diabetes* | Prospective cohort study | 229 | 13-19 | Hungary | “(…) a capacity of a dynamic system that helps individuals to overcome the negative effects, recover from adverse circumstances while maintaining normal development. It is not a quality that is always present in every situation, but a process of harnessing new and existing resources to maintain well-being during and after any stressor.” | (A. S. Masten, 2014),(Rosenberg & Yi-Frazier, 2016) | **●** PedsQL Diabetes module  **●** The Neil and Dias Resilience Scale | |
| (McGavock et al., 2018)  *Type 2 Diabetes* | Cross-sectional | 162 | 10-25 | Canada | “(…) a hedge against poor mental health” | (Dray et al., 2017) | **●** Resilience Scale for Children and Adolescents (RSCA)  **●** Patient-Based Assessment and Counseling for Physical Activity and Nutrition-Adolescent assessment forms (PACE-Adolescent) | |
| (Araia et al., 2020)  *Type 1 Diabetes* | Cross-sectional | 477 | 16 ± 2 | Australia | No definition | NA | **●** Diabetes Eating Problem Survey-Revised (DEPS-R)  **●** Diabetes Strengths and Resilience measure for adolescents (DSTAR ) | |
| (Shapiro et al., 2021)  *Type 1 Diabetes* | Longitudinal | 264 | 14-18 | USA | “Resilience refers to achieving positive emotional, behavioral, or health outcomes in the face of adversity, such as managing a complex medical condition. Resilience processes refer to protective skills and assets that contribute to optimal health and psychological outcomes when exposed to risk or adversity. Resilience processes for youth with type 1 diabetes are multi-faceted, spanning individual, familial, and contextual systems, and some can be modified through intervention to improve resilience.” | (Hilliard et al., 2013) | **●** The Coping Efficacy Scale  **●** The Social Problem-Solving Inventory – Revised (SPSI-R)  **●** Automatic Thoughts Questionnaire (ATQ)  **●** Hopelessness Scale for Children (HSC)  **●** Diabetes Family Conflict Scale-Revised (DFCS-R) | |
| **Other or combination of chronic diseases (for *diagnosis* see column author)** | | | | | | | | |
| (Rosenberg et al., 2014)  *Type 1 Diabetes, cancer* | RCT | 30 | 12-25 | USA | “(…) a universal construct describing an individual’s capacity to maintain psychological and/or physical well-being in the face of stress and is a good candidate to buffer the negative impact of serious illness among multiple populations of adolescents and young adults.” | (J. E. Haase, 2004),(Southwick & Charney, 2012) | **●** Connor-Davidson resiliency questionnaire (CD-RISC-10) | |
| (Moreira et al., 2015)  *Chronic Kidney Disease* | Cross-sectional | 28 | 9-18 | Brazil | “Resilience refers to an adaptive behavior associated with either internal states of well-being or adequate functioning in the environment and can be also defined as “a dynamic, multidimensional process, which results in positive adaptation in adverse contexts”.” | (Luthar et al., 2000) | **●** The Wagnild and Young Resilience Scale (RS) | |
| (Santos et al., 2016)  *Type 1 Diabetes, allergic diseases, neurological diseases* | Cross-sectional | 135 | 11-17 | Portugal | No definition | NA | **●** Healthy Kids Resilience Assessment Module  **●** Connectedness to school: Scale of Satisfaction with Social Support (SSSS) | |
| (S. Lee et al., 2020)  *Congenital Heart Disease, Leukemia* | Cross-sectional | 175 | 12-20 | South Korea | “(…) the process of effectively negotiating, adapting to, or managing a significant source of stress or trauma.” | (Windle, 2011) | **●** The Wagnild and Young Resilience Scale (RS) | |
| (Verma & Rohan, 2020)  *Various chronical diseases* | Cross-sectional | 50 | 13-21 | USA | “(…) a measure of adaptability to stress and ability to overcome challenges.” | (Seligman, 2011), (Luthar et al., 2000), among others | **●** Self-Management and Transition to Adulthood with Rx (STARx)  **●** Connor-Davidson resiliency questionnaire (CD-RISC-25) | |
| (Biernacka et al., 2021)  *Liver or Renal transplantation* | Cross-sectional | 96 | 12-18 | Switzerland | “Psychological resilience is mentioned among the various psychological resources facilitating beneficial adaptation to illness. Resilience is revealed in the context of coping with negative life events and difficulties. In this sense, resilience can be viewed as an indicator of mental strength. Thanks to it, despite adversities, a person can develop and maintain mental health. (…) When it comes to a chronic illness, psychological resilience is a resource that promotes adaptation to the circumstances of the illness and the limitations generated by it.” | (Rutter, 2012),(Kim, G.M. Lim, J.Y. Kim, E.J. Park, 2019) | **●** Resilience Assessment Scale for Children and Adolescents (SPP-18) | |
| (Kully-Martens et al., 2021)  *Fetal Alcohol Spectrum Disorder* | Cross-sectional | 19 | 13-23 | Canada | “Resilience is a phenomenon defined by competent (or developmentally typical) adaptation  amidst adversity.” | (A. Masten & Cicchetti, 2016) | **●** Behavioral Assessment System for Children (BASC-2)  **●** Child and Youth Resilience Measure (CYRM-28) | |
| (Tomlinson et al., 2021)  *Functional gastrointestinal disorders or organic gastrointestinal disorders* | Cross-sectional | 98 | 8-17 | Canada | (…) protective factor model of resilience in which personal assets serve as buffers between risk factors and negative outcomes (…). | (Fergus & Zimmerman, 2005) | **●** Pediatric Quality of Life Inventory (PedsQL)  **●** Youth Life Orientation Test (YLOT), optimism subscale  **●** Child Self-Efficacy Scale (CSES) | |

| **Legend** | NA = not applicable; NR = not reported; nfs = not further specified; RCT = Randomized Controlled Trial  *no validated questionnaire; ¥ this is not a questionnaire |
| --- | --- |

**References**

Adibsereshki, N., Hatamizadeh, N., Kazemnejad, A., & Sajedi, F. (2021). Resilience intervention to strengthen self-regulation in adolescent students with hearing loss. *European Journal of Mental Health*, *16*(2), 76–98. https://doi.org/10.5708/EJMH.16.2021.2.4

Araia, E., King, R. M., Pouwer, F., Speight, J., & Hendrieckx, C. (2020). Psychological correlates of disordered eating in youth with type 1 diabetes: Results from diabetes MILES Youth—Australia. *Pediatric Diabetes*, *21*(4), 664–672. https://doi.org/10.1111/pedi.13001

Arruda, M. A., Arruda, R., Landeira-Fernandez, J., Anunciação, L., & Bigal, M. E. (2021). Resilience and vulnerability in adolescents with primary headaches: A cross-sectional population-based study. *Headache*, *61*(3), 546–557. https://doi.org/10.1111/head.14078

Bahryni, S., Bermas, H., & Tashvighi, M. (2016). The self-efficacy forecasting based on hope to life and resiliency in adolescents suffering from cancer. *Biomedical and Pharmacology Journal*, *9*(3), 1147–1156.

Beeckman, M., Hughes, S., van Ryckeghem, D., van Hoecke, E., Dehoorne, J., Joos, R., & Goubert, L. (2019). Resilience factors in children with juvenile idiopathic arthritis and their parents: The role of child and parent psychological flexibility. *Pain Medicine (United States)*, *20*(6), 1120–1131. https://doi.org/10.1093/pm/pny181

Biernacka, M., Jakubowska-Winecka, A., & Kalicí nski, P. (2021). *Influence of Parental Attitudes on Formation of Psychological Resilience and Adherence to Medical Regime in Adolescents after Liver or Renal Transplantation*. https://doi.org/10.3390/children

Block, J. H., & Block, J. (1980). *The role of ego-control and Ego- resiliency in the origination of behavior* (Vol. 13). WA Collings (red.). The Minnesota Symposia on Child Psychology.

Bonanno, G. A., Westphal, M., & Mancini, A. D. (2011). Resilience to Loss and Potential Trauma. *Annual Review of Clinical Psychology*, *7*(1), 511–535.

Borinsky, S., Gaughan, J. P., & Feldman-Winter, L. (2019). Perceived overweight/obesity, low resilience, and body size dissatisfaction among adolescents. *Obesity Research and Clinical Practice*, *13*(5), 448–452. https://doi.org/10.1016/j.orcp.2019.08.002

Buehler, C. (2020). Family Processes and Children’s and Adolescents’ Well-Being. *Journal of Marriage and Family*, *82*(1), 145–174. https://doi.org/10.1111/jomf.12637

Carlsen, K., Haddad, N., Gordon, J., Phan, B. L., Pittman, N., Benkov, K., Dubinsky, M. C., & Keefer, L. (2017). Self-efficacy and Resilience Are Useful Predictors of Transition Readiness Scores in Adolescents with Inflammatory Bowel Diseases. *Inflammatory Bowel Diseases*, *23*(3), 341–346.

Chmitorz, A., Kunzler, A., & Helmreich, I. (2018). Intervention studies to foster resilience: a systematic review and proposal for a resilience framework in future intervention studies. *Clin Psychol Rev*, *59*, 78–100.

Chung, J. O. K., Li, W. H. C., Cheung, A. T., Ho, L. L. K., Xia, W., Chan, G. C. F., & Lopez, V. (2021). Relationships among resilience, depressive symptoms, self-esteem, and quality of life in children with cancer. *Psycho-Oncology*, *30*(2), 194–201. https://doi.org/10.1002/pon.5548

Compas, B. E. (2006). Psychobiological processes of stress and coping: Implications for resilience in children and adolescents - Comments on the papers of Romeo & McEwen and Fisher et al. *Annals of the New York Academy of Sciences*, *1094*, 226–234.

Connor, K. M., & Davidson, J. R. T. (2003). Development of a new Resilience scale: The Connor-Davidson Resilience scale (CD-RISC). *Depression and Anxiety*, *18*(2), 76–82.

Cousins, L., Kalapurakkel, S., Cohen, L., & Simons, L. (2015). Topical review: Resilience resources and mechanisms in pediatric chronic pain. *J Pediatr Psychol*, *40*(9), 840–845.

Cui, C., Shuang-zi, L., Cheng, W. jin, & Wang, T. (2022). Mediating effects of coping styles on the relationship between family resilience and self-care status of adolescents with epilepsy transitioning to adult healthcare: A cross-sectional study in China. *Journal of Pediatric Nursing*, *63*, 143–150. https://doi.org/10.1016/j.pedn.2021.11.021

Davis, D. (2004). *Child Development: A Practitioner’s Guide* (2nd ed.). The Guilford Press.

Davydov, D. M., Stewart, R., Ritchie, K., & Chaudieu, I. (2010). Resilience and mental health. *Clinical Psychology Review*, *30*(5), 479–495. https://doi.org/10.1016/j.cpr.2010.03.003

Dent, M. (2008). *Promoting resilience in the millennial adolescent: The lighthouse model.*

Diener, E., Oishi, S., & Lucas, R. E. (2003). Personality, Culture, and Subjective Well-Being: Emotional and Cognitive Evaluations of Life. *Annual Review of Psychology*, *54*(1), 403–425.

Dray, J., Bowman, J., Campbell, E., & et al. (2017). Systematic Review of Universal Resilience-Focused Interventions Targeting Child and Adolescent Mental Health in the School Setting. *Journal of the American Academy of Child and Adolescent Psychiatry*, *56*(10), 813–824.

Fee, R. J., & Hinton, V. J. (2011). Resilience in children diagnosed with a chronic neuromuscular disorder. *Journal of Developmental and Behavioral Pediatrics : JDBP*, *32*(9), 644–650.

Fergus, S., & Zimmerman, M. (2005). ADOLESCENT RESILIENCE: A Framework for Understanding Healthy Development in the Face of Risk. *Annu. Rev. Public Health*, *26*, 399–419.

Furniss, D., Barber, N., Lyons, I., & al, E. (2014). Unintentional non-Adherence: Can a spoon full of resilience help the medicine go down? *BMJ Quality and Safety*, *23*(2), 95–98.

Gheshlagh, R., Ebadi, A., & Dalvandi, A. (2016). A systematic study of resilience in patients with chronic physical diseases. *Nurs Midwifery Stud*, *6*(2).

Gillham, J. E., Hamilton, J., Freres, D. R., & al, E. (2006). Preventing depression among early adolescents in the primary care setting: A randomized controlled study of the Penn Resiliency Program. *Journal of Abnormal Child Psychology*, *34*(2), 203–219.

Gmuca, S., Sonagra, M., Xiao, R., Miller, K. S., Thomas, N. H., Young, J. F., Weiss, P. F., Sherry, D. D., & Gerber, J. S. (2021). Suicidal risk and resilience in juvenile fibromyalgia syndrome: a cross-sectional cohort study. *Pediatric Rheumatology*, *19*(1). https://doi.org/10.1186/s12969-020-00487-w

Goubert, L., & Trompetter, H. (2018). Towards a science and practice of resilience in the face of pain. *Eur J Pain*, *21*(8), 1301–1315.

Haase, J. E. (2004). The adolescent resilience model as a guide to interventions. *Journal of Pediatric Oncology Nursing*, *21*(5), 289–299. https://doi.org/10.1177/1043454204267922

Haase, J. E., Kintner, E. K., Monahan, P. O., & Robb, S. L. (2014). The resilience in illness model, part 1: exploratory evaluation in adolescents and young adults with cancer. *Cancer Nursing*, *37*(3), E1-12.

Haase, J., Heiney, S., Ruccione, K., & Stutzer, C. (1999). Research triangulation to derive meaning-based quality-of-life theory: adolescent resilience model and instrument development. *Int J Cancer Suppl*, *12*, 125–131.

Hilliard, M. E., Holmes, C. S., Chen, R., Maher, K., Robinson, E., & Streisand, R. (2013). Disentangling the roles of parental monitoring and family conflict in adolescents’ management of type 1 diabetes. *Health Psychology*, *32*(4), 388–396. https://doi.org/10.1037/a0027811

Hood, K. K., Iturralde, E., Rausch, J., & et al. (2018). Preventing diabetes distress in adolescents with type 1 diabetes: Results 1 year after participation in the STePS program. *Diabetes Care*, *41*(8), 1623–1630.

Huang, H. R., Chen, C. W., Chen, C. M., Yang, H. L., Su, W. J., Wang, J. K., & Tsai, P. K. (2018). A positive perspective of knowledge, attitude, and practices for health-promoting behaviors of adolescents with congenital heart disease. *European Journal of Cardiovascular Nursing*, *17*(3), 217–225.

Hunter, A., & Chandler, G. (1999). Adolescent resilience. *IMAGE: Journal of Nursing Scholarship*, *31*(3243–247).

Huston, S. A., Bloun, R. L., & Heidsec, T. (2016). Resilience, emotion processing and emotionexpression among youth with type 1 diabetes. *Pediatric Diabetes*, *17*, 623–631.

Iacoviello, B. M., & Charney, D. S. (2014). Psychosocial facets of resilience: implications for preventing posttrauma psychopathology, treating trauma survivors, and enhancing community resilience. *European Journal of Psychotraumatology*, *5*, 1–10.

Im, Y. J., & Kim, D. H. (2012). Factors associated with the resilience of school-aged children with atopic dermatitis. *Journal of Clinical Nursing*, *21*(1), 80–88.

McGavock, J., Durksen, A., Wicklow, B., Malik, S., Sellers, E., Blydt-Hansen, T., Chateau, D., & Dart, A. (2018). Determinants of Readiness for Adopting Healthy Lifestyle Behaviors Among Indigenous Adolescents with Type 2 Diabetes in Manitoba, Canada: A Cross-Sectional Study. *Obesity*, *26*(5), 910–915.

Jaser, S. S., & White, L. E. (2011). Coping and resilience in adolescents with type 1 diabetes. *Child: Care, Health and Development*, *37*(3), 335–342.

Johnson, M. O., Catz, S. L., Remien, R. H., Rotheram-Borus, M. J., Morin, S. F., Charlebois, E., Gore-Felton, C., Goldsten, R. B., Wolfe, H., Lightfoot, M., & Chesney, M. A. (2003). Theory-Guided, Empirically Supported Avenues for Intervention on HIV Medication Nonadherence: Findings from the Healthy Living Project Downloaded. *Aids Patient Care*, *17*(12).

Kaewkong, P., Boonchooduang, N., Charoenkwan, P., & Louthrenoo, O. (2020). Resilience in adolescents with thalassemia. *Pediatric Hematology and Oncology*, *38*(2), 124–133. https://doi.org/10.1080/08880018.2020.1821140

Kim, D. H., & Im, Y. J. (2014). Resilience as a protective factor for the behavioral problems in school-aged children with atopic dermatitis. *Journal of Child Health Care : For Professionals Working with Children in the Hospital and Community*, *18*(1), 47–56.

Kim, D. H., & Yoo, I. Y. (2010). *Factors associated with resilience of school age children with cancer*. *46*(7), 431–436.

Kim, G.M. Lim, J.Y. Kim, E.J. Park, S. M. (2019). Resilience of patients with chronic diseases : A systematic review. *Health Soc Care Community*, *27*, 797–807.

Kully-Martens, K., Pei, J., McNeil, A., & Rasmussen, C. (2021). Resilience Resources and Emotional and Behavioral Functioning Among Youth and Young Adults with Fetal Alcohol Spectrum Disorder. *International Journal of Mental Health and Addiction*. https://doi.org/10.1007/s11469-021-00652-6

Lau, N., Yi-Frazier, J. P., Bona, K., Baker, K. S., McCauley, E., & Rosenberg, A. R. (2020). Distress and resilience among adolescents and young adults with cancer and their mothers: An exploratory analysis. *Journal of Psychosocial Oncology*, *38*(1), 118–124. https://doi.org/10.1080/07347332.2019.1656317

LeBovidge, J. S., Strauch, H., Kalisch, L. A., & Al, E. (2009). Assessment of psychological distress among children and adolescents with food allergy. *J ALLERGY CLIN IMMUNOL*, *124*(6). https://doi.org/10.1016/j.jpsychores.2014.10.005

Lee, J. Y., Jeong, D. C., Chung, N. G., & Lee, S. (2019). The effects of illness cognition on resilience and quality of life in korean adolescents and young adults with leukemia. *Journal of Adolescent and Young Adult Oncology*, *8*(5), 610–615. https://doi.org/10.1089/jayao.2018.0152

Lee, S., Chung, N. G., & Choi, J. Y. (2020). Comparison of resilience and quality of life between adolescent blood cancer survivors and those with congenital heart disease: A cross sectional study. *Health and Quality of Life Outcomes*, *18*(1), 1–7. https://doi.org/10.1186/s12955-020-01487-w

Lee, S., Kim, S., & Young Choi, J. (2014). Coping and resilience of adolescents with congenital heart disease. *The Journal of Cardiovascular Nursing*, *29*(4), 340–346.

Lee, S., Lee, J., & Choi, J. Y. (2017). The effect of a resilience improvement program for adolescents with complex congenital heart disease. *European Journal of Cardiovascular Nursing : Journal of the Working Group on Cardiovascular Nursing of the European Society of Cardiology*, *16*(4), 290–298.

Li, M. K., Patel, B. P., Chu, L., Strom, M., & Hamilton, J. K. (2022). Investigating resilience and its association with stress, anthropometrics, and metabolic health in adolescents with obesity: a pilot study. *Psychology, Health and Medicine*. https://doi.org/10.1080/13548506.2022.2059094

Lukacs, A., Mayer, K., Sasvari, P., & Al, E. (2018). Health-related quality of life of adolescents with type 1 diabetes in the context of resilience. *Pediatric Diabetes*, *19*(8), 1481–1486.

Luthar, S. S., Cicchetti, D., & Becker, B. (2000). The Construct of Resilience: A Critical Evaluation and Guidelines for Future Work. *Child Dev.*, *71*(3), 543–562.

Mandleco, B. L., & Peery, J. C. (2000). An organizational framework for conceptualizing resilience in children. *Child Adolesc Psychiatr Nurs*, *13*, 99–111.

Masten, A., & Cicchetti, D. (2016). Resilience in development: Progress and transformation. . In *Developmental psychopathology* (3rd ed., Vol. 4). John Wiley & Sons.

Masten, A. S. (2001). Ordinary magic. Resilience processes in development. *AM Psychol*, *56*(3), 227–238.

Masten, A. S. (2007). Resilience in developing systems: Progress and promise as the fourth wave rises. *Development and Psychopathology*, *19*(3), 921–930.

Masten, A. S. (2014). Global Perspectives on Resilience in Children and Youth. *Child Development*, *85*(1), 6–20.

Masten, A. S., & O’Connor, M. J. (1988). Vulnerability, stress and resilience in the early development of a high risk child. *American Academy of Child and Adolescent Psychiatry*, *28*, 274–278.

Moreira, J. M., Bouissou Morais Soares, C. M., Teixeira, A. L., Simoes e Silva, A. C., & Kummer, A. M. (2015). Anxiety, depression, resilience and quality of life in children and adolescents with pre-dialysis chronic kidney disease. *Pediatric Nephrology (Berlin, Germany)*, *30*(12), 2153–2162.

Nabors, L. A., Graves, M. L., Fiser, K. A., & Merianos, A. L. (2021). Family resilience and health among adolescents with asthma only, anxiety only, and comorbid asthma and anxiety. *Journal of Asthma*, *58*(12), 1599–1609. https://doi.org/10.1080/02770903.2020.1817939

Parviniannasab, A. M., Rakhshan, M., Momennasab, M., Soltanian, M., Rambod, M., & Akbarzadeh, M. (2022). The mediating role of Courageous coping in the relations between spirituality and social support with resilience among adolescents with hemophilia. *Clinical Child Psychology and Psychiatry*, *27*(4), 1141–1154. https://doi.org/10.1177/13591045211055081

Rainone, N., Chiodi, A., Lanzillo, R., & Al, E. (2017). Affective disorders and Health-Related Quality of Life (HRQoL) in adolescents and young adults with Multiple Sclerosis (MS): The moderating role of resilience. *Quality of Life Research*, *26*(3), 727–736.

Rassart, J., Luyckx, K., Goossens, E., & Al, E. (2016). A big five personality typology in adolescents with congenital heart disease: Prospective associations with psychosocial functioning and perceived health. *International Journal of Behavioral Medicine*, *23*(3), 310–318.

Rew, L., & Horner, S. D. (2003). Youth resilience framework for reducing health-risk behaviors in adolescents. *Journal of Pediatric Nursing*, *18*(6), 379–388. https://doi.org/10.1016/S0882-5963(03)00162-3

Robb, S. L., Burns, D. S., Stegenga, K. A., Haut, P. R., Monahan, P. O., Meza, J., Stump, T. E., Cherven, B. O., Docherty, S. L., & Hendricks-Ferguson, V. L. (2014). Randomized clinical trial of therapeutic music video intervention for resilience outcomes in adolescents/young adults undergoing hematopoietic stem cell transplant: a report from the Children’s Oncology Group. *Cancer*, *120*(6), 909–917.

Rosenberg, A. R., Bradford, M. C., McCauley, E., Curtis, J. R., Wolfe, J., Baker, K. S., & Yi-Frazier, J. P. (2018). Promoting resilience in adolescents and young adults with cancer: Results from the PRISM randomized controlled trial. *Cancer*, *124*(19), 3909–3917.

Rosenberg, A. R., & Yi-Frazier, J. P. (2016). Commentary: Resilience defined: An alternative perspective. *Journal of Pediatric Psychology*, *41*(5), 506–509. https://doi.org/10.1093/jpepsy/jsw018

Rosenberg, A. R., Yi-Frazier, J. P., Eaton, L., Wharton, C., Cochrane, K., Pihoker, C., Baker, K. S., & McCauley, E. (2014). Promoting resilience in stress management: A pilot study of a novel resilience-promoting intervention for adolescents and young adults with serious illness. *Journal of Pediatric Psychology*, *40*(9), 992–999.

Ruff, R. R., Sischo, L., & Broder, H. (2016). Resiliency and socioemotional functioning in youth receiving surgery for orofacial anomalies. *Community Dentistry and Oral Epidemiology*, *44*(4), 371–380.

Rutter, M. (2012). Resilience as a dynamic concept. *Development and Psychopathology*, *24*(2), 335–344. https://doi.org/10.1017/S0954579412000028

Santos, T., de Matos, M. G., Marques, A., Simoes, C., Leal, I., & Machado, M. do C. (2016). Adolescent’s subjective perceptions of chronic disease and related psychosocial factors: highlights from an outpatient context study. *BMC Pediatrics*, *16*(1), 211.

Schwartz, L. A., & Brumley, L. D. (2017). What a Pain: The Impact of Physical Symptoms and Health Management on Pursuit of Personal Goals Among Adolescents with Cancer.pdf. *Journal of Adolescent and Young Adult Oncology*, *6*(1).

Seligman, M. (2011). Building resilience. *Harv. Bus. Rev*, *89*, 100–106.

Shapiro, J. B., Bryant, F. B., Holmbeck, G. N., Hood, K. K., & Weissberg-Benchell, J. (2021). Do baseline resilience profiles moderate the effects of a resilience-enhancing intervention for adolescents with type I diabetes? *Health Psychology*, *40*(5), 337–346. https://doi.org/10.1037/hea0001076

Sharp, C., Penner, F., Marais, L., & Lochner, S. (2018). School connectedness as psychological resilience factor in children affected by HIV/AIDS. *AIDS CARE*, *30*(4), 34–41.

Sharp, K., Willard, V. W., Okado, Y., Tillery, R., Barnes, S., Long, A., & Phipps, S. (2015). *Profiles of connectedness: Processes of resilience and growth in children with cancer*. *40*(9), 904–913. http://ovidsp.ovid.com/ovidweb.cgi?T=JS&PAGE=reference&D=psyc12&NEWS=N&AN=2015-45524-008

Simon, K., Barakat, L. P., Patterson, C. A., & Al, E. (2009). *Symptoms of depression and anxiety in adolescents with sickle cell disease: The role of intrapersonal characteristics and stress processing variables*. *40*(2), 317–330.

Southwick, S. M., Bonanno, G. A., Masten, A. S., Panter-Brick, C., & Yehuda, R. (2014). Resilience definitions, theory, and challenges: Interdisciplinary perspectives. *European Journal of Psychotraumatology*, *5*. https://doi.org/10.3402/ejpt.v5.25338

Southwick, S. M., & Charney, D. S. (2012). The Science of Resilience: Implications for the Prevention and Treatment of Depression. *Science*, *338*(October), 79–83.

Southwick, S. M., Vythilingam, M., & Charney, D. S. (2005). The Psychobiology of Depression and Resilience to Stress: Implications for Prevention and Treatment. *Annual Review of Clinical Psychology*, *1*(1), 255–291.

Stewart, D. E., & Yuen, T. (2011). A Systematic Review of Resilience in the Physically Ill. *Psychosomatics*, *52*(3), 199–209. https://doi.org/10.1016/j.psym.2011.01.036

Sturgeon, J., & Zautra, A. (2010). Resilience: A new paradigm for adaptation to chronic pain. *Curr Pain Headache Rep*, *14*(2), 105–114.

Tang, Y., Chen, W., Li, J., Deng, Y., Liu, S., Zhou, X., Xie, J., Zhan, C., & Li, X. (2022). A disease-targeted picture book for children with Henoch-Schonlein purpura nephritis: A quasi-experimental study. *Journal of Renal Care*. https://doi.org/10.1111/jorc.12451

Tomlinson, R. M., Bax, K. C., Ashok, D., & McMurtry, C. M. (2021). Health-related quality of life in youth with abdominal pain: An examination of optimism and pain self-efficacy. *Journal of Psychosomatic Research*, *147*. https://doi.org/10.1016/j.jpsychores.2021.110531

Traub, F., & Boynton-Jarett, R. (2017). Modifiable  Resilience Factors to Childhood Adversity for  Clinical Pediatric Practice. *Pediatrics*, *139*(5).

Verma, T., & Rohan, J. (2020). Examination of transition readiness, medication adherence, and resilience in pediatric chronic illness populations: A pilot study. *International Journal of Environmental Research and Public Health*, *17*(6), 4–14. https://doi.org/10.3390/ijerph17061905

Wagnild, G. M., & Young, H. M. (1993). Development and psychometric evaluation of the Resilience Scale. *Journal of Nursing Measurement*, *1*(2), 165–178.

Wallander, J. L., & Varni, J. W. (1998). Effects of pediatric chronic physical disorders on child and family adjustment. *Journal of Child Psychology and Psychiatry and Allied Disciplines*, *39*(1), 29–46.

Walsh, F. (2003). Family resilience: A framework for clinical practice. *Family Process*, *42*(1), 1–18. https://doi.org/10.1111/j.1545-5300.2003.00001.x

Walsh, F. (2016). Family resilience: a developmental systems framework. *European Journal of Developmental Psychology*, *13*(3), 313–324. https://doi.org/10.1080/17405629.2016.1154035

Werner, E. E. (1989). Children of the Garden Island. *Scientific American*, *260*(4), 106–111.

Whiteley, L., Brown, L. K., Mena, L., & et al. (2019). Enhancing health among youth living with HIV using an iPhone game. *AIDS Care*.

Willard, V. W., Russell, K. M., Long, A., & Al, E. (2018). The impact of connectedness on social functioning in youth with brain tumors. *Pediatric Blood and Cancer*, *66*(5).

Windle, G. (2011). What is resilience? A review and concept analysis. *Reviews in Clinical Gerontology*, *21*(2), 152–169. https://doi.org/10.1017/S0959259810000420

Wright, L. A., Cohen, L. L., Gise, J., Shih, S., Sil, S., & Carter, S. (2021). Pain and QOL in pediatric sickle cell disease: Buffering by resilience processes. *Journal of Pediatric Psychology*, *46*(8), 1015–1024. https://doi.org/10.1093/jpepsy/jsab034

Wu, G., Feder, A., Cohen, H., & et al. (2013). Understanding resilience. *Front Behav Neurosci*, *7*.

Wu, L. M., Sheen, J. M., Shu, H. L., & et al. (2013). Predictors of anxiety and resilience in adolescents undergoing cancer treatment. *Journal of Advanced Nursing*, *69*(1), 158–166.

Wu, W., Chang, J., Tsai, S., & Liang, S. (2018). Assessing Self-concept as a Mediator between Anger and Resilience in Adolescents with Cancer in Taiwan. *Cancer Nursing*, *41*(3), 210–217.

Wu, Z., Liu, Y., Li, X., & Li, X. (2016). Resilience and Associated Factors among Mainland Chinese Women Newly Diagnosed with Breast Cancer. *PLoS ONE*, *11*(12).

Yi, J. P., Vitaliano, P. P., Smith, R. E., & et al. (2008). *The role of resilience on psychological adjustment and physical health in patients with diabetes*. *13*(Pt 2), 311–325.

Yi-Frazier, J. P., Yaptangco, M., Semana, S., Buscaino, E., Thompson, V., Cochrane, K., Tabile, M., Alving, R., & Rosenberg, A. R. (2015). The association of personal resilience with stress, coping, and diabetes outcomes in adolescents with type 1 diabetes: variable- and person-focused approaches. *Journal of Health Psychology*, *20*(9), 1196–1206.

Zimmerman, K., May, B., Barnes, K., Arynchyna, A., Chagoya, G., Alford, E. N., Wessinger, C. A., Dreer, L., Aban, I., Johnston, J. M., Rozzelle, C., Blount, J. P., & Rocque, B. G. (2021). Post-Traumatic Stress Symptoms in Caregivers and Children with Hydrocephalus. *World Neurosurgery*, *148*, e66–e73. https://doi.org/10.1016/j.wneu.2020.12.008
