# Supplementary material for: Defining and Measuring Resilience in Children with a Chronic Disease: a Scoping Review
Source: Advers Resil Sci. 2023 Apr 10;4(2):105–23. doi: 10.1007/s42844-023-00092-2 (PMC10088629; doi:10.1007/s42844-023-00092-2)
Supplement: Supplementary file 3 — Supplementary file3 (DOCX 111 KB) [file 42844_2023_92_MOESM3_ESM.docx]

| **Instruments** | **Previously used in youth with diagnosis*** | **Items** | **Response** | **Range ^a^** | **Instrument measures** | | |
| --- | --- | --- | --- | --- | --- | --- | --- |
|  |  |  |  |  | **Personal trait** | **Psychosocial functioning** | **Disease-related outcomes** |
| **The Adolescent Self-Regulatory Inventory (ASRI)** (Dias et al., 2014) | Hearing loss | 36 | 5-point Likert scale | 36-180 |  |  |  |
|  |  |  |  |  | **●** Short term self-regulation  **●** Long term self-regulation | | |
| **Behavior Assessment System for Children 2^nd^ edition (BASC-2)** (Reynolds & Kamphaus, 2004) | Food allergy  SCD  Asthma | 36 | 4-point Likert scale | 36-144 |  |  |  |
|  |  |  |  |  | **●** Adaptive skills  **●** Behavioral symptoms index  **●** Externalizing problems  **●** Internalizing problems  **●** School problems | | |
| **Beck Youth Inventory for Emotional and Social Impairment (2nd edition)** (Beck et al., 2005) | Orofacial anomalies | 100 | NR | NR |  |  |  |
|  |  |  |  |  | **●** Depression  **●** Anxiety  **●** Concept of self | | |
| **Benefit Finding/Burden Scale for Children (BBSC)** (Currier et al., 2009) | Cancer | 20 | 5-point Likert scale | NR |  |  |  |
|  |  |  |  |  | **●** Potential benefits or burdens of illness | | |
| **Benefit Finding Scale for Children (BFSC)** (Phipps et al., 2007) | T1D | 10 | 5-point Likert scale | NR |  |  |  |
|  |  |  |  |  | **●** Benefit finding in illness | | |
| **Child Behavior Checklist ((K-)CBCL)** (Achenbach, 1991) | DMD, atopic dermatitis | 118 | 3-point Likert scale | 0-236 |  |  |  |
|  |  |  |  |  | **●** Emotional problems  **●** Behavioral problems | | |
| **Connor-Davidson resiliency questionnaire (CD-RISC-10)** (Campbell-Sills & Stein, 2007) | IBD, cancer, T1D | 10 | 5-point Likert scale | 0-40 |  |  |  |
|  |  |  |  |  | **●** Perceived ability to tolerate experiences  **●** Humor  **●** Self-efficacy | | |
| **Contour Drawing Rating Scale (CDRS)** (Thompson & Gray, 1995) | Obesity | 2 | 9 options | NA |  |  |  |
|  |  |  |  |  | **●** Body size dissatisfaction | | |
| **Center for Epidemiologic Studies Depression Scale (CESDS)** (Bouma et al., 1995) | CHD | 20 | 4-point Likert scale | 0-60 |  |  |  |
|  |  |  |  |  | **●** Depressive symptoms | | |
| **Chronic illness children’s resilience scale (CICRS)** (Kim & Yoo, 2010a) | Henoch Schonlein purpura nephritis | 32 | 4-point Likert scale | 32-128 |  |  |  |
|  |  |  |  |  | **●** Interpersonal characteristics  **●** Coping characteristics  **●** Intrapersonal relationships | | |
| **Diabetes Eating Problem Survey-Revised (DEPS-R)** (Markowitz et al., 2010) | T1D | 16 | 6-point Likert scale | 0-80 |  |  |  |
|  |  |  |  |  | **●** Disordered eating | | |
| **Diabetes acceptance** (Huston et al., 2016)****** | T1D | 3 | NR | NR |  |  |  |
|  |  |  |  |  | **●** Illness acceptance | | |
| **Diabetes, fitting in** (Huston et al., 2016)****** | T1D | 5 | NR | NR |  |  |  |
|  |  |  |  |  | **●** Fitting in with illness | | |
| **Diabetes, comfort in adjusting for diabetes in public** (Huston et al., 2016)****** | T1D | 3 | NR | NR |  |  |  |
|  |  |  |  |  | **●** Comfort in adjusting for illness in public | | |
| **Family resilience (NSCH)** (Nabors et al., 2021) | Asthma | 4 | 4-point Likert scale | 4-16 |  |  |  |
|  |  |  |  |  | ﻿**●** Family flourishing | | |
| **General self-efficacy questionnaire (GFE-10)** (Bahryni et al., 2016) | Cancer | 10 | 4-point Likert scale | 10-40 |  |  |  |
|  |  |  |  |  | **●** General self-efficacy  **●** Social self-efficacy | | |
| **Haase Adolescent Resilience in Illness Scale (HARIS)** (Haase et al., 2014) | Cancer, CHD, HSCT | 13 | 6-point Likert scale | 13-78 |  |  |  |
|  |  |  |  |  | **●** Self-esteem  **●** Sense of mastery  **●** Positive expectation | | |
| **Health-related Hindrance Inventory** (Schwartz & Drotar, 2009) | Cancer | 10 | 7 point-Likert scale | 0-60 |  |  |  |
|  |  |  |  |  | **●** Impact of health on personal goals | | |
| **Leuven Knowledge Questionnaire for Congenital Heart Disease (LKQCHD)** (Yang et al., 2012) | CHD | 34 (female)  31 (male) | 3-point Likert scale | NR |  |  |  |
|  |  |  |  |  | **●** Level of disease knowledge | | |
| **Metabolic control (HbA1c)** (Jaser & White, 2011)**¥** | T1D | NA | NA | NA |  |  |  |
|  |  |  |  |  | **●** Metabolic control | | |
| **Problem Areas in Diabetes Scale (PAID)** (Welch et al., 1997) | T1D | 20 | 5-point Likert scale | 0-80 |  |  |  |
|  |  |  |  |  | **●** Illness related distress | | |
| **Positive and Negative Affect Scale for Children (PANAS-c)** (Laurent et al., 1999) | JIA | 27 | 5-point Likert scale | 5-135 |  |  |  |
|  |  |  |  |  | **●** Positive and negative affect | | |
| **Pediatric Quality of Life Inventory (PedsQL)** (J. Varni et al., 1999) | CHD | 23 | 5-point Likert scale | 0-92 |  |  |  |
|  |  |  |  |  | **●** Physical functioning  **●** Emotional functioning  **●** Social functioning  **●** School-related functioning | | |
| **Pediatric Quality of Life Inventory (****PedsQL)** (J. W. Varni et al., 1999)  ***Cardiac module***  ***Diabetes module*** | T1D | 27  33 | 5-point Likert scale | 0-108  0-132 |  |  |  |
|  |  |  |  |  | **●** Disease-related quality of life | | |
| **UCLA PTSD Reaction Index for DSM-IV (PTSDI)** (Pynoos et al., 1998) | Cancer | 22 | 5-point Likert scale | 0-88 |  |  |  |
|  |  |  |  |  | **●** PTSD symptoms | | |
| **Resilience measurement instrument for children with chronic illness** (Kim & Yoo, 2010b) | Atopic dermatitis, cancer | 32 | 4-point Likert scale | 32-128 |  |  |  |
|  |  |  |  |  | **●** Self-understanding  **●** Self-reliance  **●** Resourcefulness  **●** Perception of family relationships  **●** Perception of interpersonal intimacy | | |
| **The Wagnild and Young Resilience Scale (RS)** (Wagnild & Young, 1993) | CHD, CKD, T1D, cancer | 25 | 7-point Likert scale | 25-175 |  |  |  |
|  |  |  |  |  | **●** Purpose  **●** Perseverance  **●** Self-reliance  **●** Equanimity  **●** Authenticity | | |
| **Resilience Scale for Children and Adolescents (RSCA)** (Prince-Embury, 2007) | T1D, Orofacial anomalies | 64 | 5-point Likert scale | 0-256 |  |  |  |
|  |  |  |  |  | **●** Self-perception of skills and competences  **●** Emotional reactivity  **●** Sense of relatedness | | |
| **Strengths and Difficulties Questionnaire (SDQ)** (Goodman, 1997) | HIV/AIDS | 25 | 3-point Likert scale | 0-50 |  |  |  |
|  |  |  |  |  | **●** Emotional problems  **●** Behavioral problems | | |
| **Social-Emotional Assets and Resilience Scales (SEARS)** (Merell, 2011) | Cancer | 35 | 4-point Likert scale | NR |  |  |  |
|  |  |  |  |  | **●** Self-regulation  **●** Responsibility  **●** Empathy  **●** Social Competence | | |
| **Self-Management and Transition to Adulthood with Rx (STARx)** (Fenton et al., 2015) | Various chronic diseases | 18 | 5-point Likert scale | 0-90 |  |  |  |
|  |  |  |  |  | **●** Transition readiness | | |
| **Transition Readiness Assessment Questionnaire (TRAQ)** (Wood et al., 2014) | IBM | 20 | 5-point Likert scale | 20-100 |  |  |  |
|  |  |  |  |  | **●** Transition readiness | | |
| **UCLA loneliness scale** (Roberts et al., 1993) | CHD | 8 | 5-point Likert scale | 8-40 |  |  |  |
|  |  |  |  |  | **●** Loneliness | | |
| **Youth Self Report (YSR)** (Achenbach & Rescorla, 2001) | T1D | 112  20 | 3-point Likert scale | 0-224  0-40 |  |  |  |
|  |  |  |  |  | **●** Perception of competence  **●** Perception of social competence | | |

|  | Disease or therapy | CHD = Congenital Heart Disease; CKD = Chronic Kidney Disease; DMD = Duchenne Muscular Dystrophy; HIV = Human Immunodeficiency Virus; HSCT = Hematopoietic Stem Cell Transplant; IBD= Inflammatory Bowel Diseases; SCD = Sickle Cell Disease; T1D = Type 1 Diabetes. |
| --- | --- | --- |
| **Legend** | Study design | RCT = Randomized Controlled Trial |
|  | Other | NA = not applicable; NR = not reported; pr= parents reported  ¥ = this is not a questionnaire  **^a^  =** raw scores, not transformed |

**References**

Achenbach, T. M. (1991). *The Manual for the Child Behavior Checklist/4-18 and 1991 Profile*. University of Vermont, Department of Psychiatry.

Achenbach, T. M., & Rescorla, L. A. (2001). *Manual for the ASEBA School-Age Forms and Profiles*. University of Vermont, Research Centre for Children, Youth and Families.

Bahryni, S., Bermas, H., & Tashvighi, M. (2016). The self-efficacy forecasting based on hope to life and resiliency in adolescents suffering from cancer. *Biomedical and Pharmacology Journal*, *9*(3), 1147–1156.

Beck, J., Beck, A., Jolly, J., & Steer, R. (2005). *Beck youth inventories for children and adolescents*. NCS Pearson, Inc.

Bouma, J., Ranchor, A., Sanderman, R., & van Sonderen, E. (1995). *Het meten van symptomen van depressie met de CESD: een handleiding [The measurement ofsymptoms ofdepression with the CESD: a manual].*

Campbell-Sills, L., & Stein, M. B. (2007). Psychometric analysis and refinement of the connor–davidson resilience scale (CD-RISC): Validation of a 10-item measure of resilience. *Journal of Traumatic Stress*, *20*(6), 1019–1028.

Currier, J., Hermes, S., & Phipps, S. (2009). Children’s response to serious illness: Perceptions of bene-fit and burden in a pediatric cancer population. *Journal of Pediatric Psychology*, *34*(1129–1134).

Dias, P. C., del Castillo, J. A. G., & Moilanen, K. L. (2014). The Adolescent Self-Regulatory Inventory (ASRI) Adaptation to Portuguese Context. *Paidéia (Ribeirão Preto)*, *24*(58), 155–164. https://doi.org/10.1590/1982-43272458201403

Fenton, N., Ferris, M., Ko, Z., Javalkar, K., & Hooper, S. (2015). The relationship of health care transition readiness to disease-related characteristics, psychosocial factors, and health care outcomes: Preliminary findings in adolescents with chronic kidney disease. *J Pediatr Rehabil Med*, *8*, 13–22.

Goodman, R. (1997). The strengths and difficulties questionnaire: A research note. *Journal of Child Psychology and Psychiatry and Allied Disciplines*, *38*(5), 581–586. https://doi.org/10.1111/j.1469-7610.1997.tb01545.x

Haase, J. E., Kintner, E. K., Monahan, P. O., & Robb, S. L. (2014). The resilience in illness model, part 1: exploratory evaluation in adolescents and young adults with cancer. *Cancer Nursing*, *37*(3), E1-12.

Huston, S. A., Bloun, R. L., & Heidsec, T. (2016). Resilience, emotion processing and emotionexpression among youth with type 1 diabetes. *Pediatric Diabetes*, *17*, 623–631.

Jaser, S. S., & White, L. E. (2011). Coping and resilience in adolescents with type 1 diabetes. *Child: Care, Health and Development*, *37*(3), 335–342.

Kim, D. H., & Yoo, I. Y. (2010a). Development of a Questionnaire to Measure Resilience in Children with Chronic Diseases. *Journal of Korean Academy of Nursing*, *40*(2), 236. https://doi.org/10.4040/jkan.2010.40.2.236

Kim, D. H., & Yoo, I. Y. (2010b). Development of a Questionnaire to Measure Resilience in Children with Chronic Diseases. *Journal of Korean Academy of Nursing*, *40*(2), 236.

Laurent, J., Catanzaro, J., Joiner, T., & et al. (1999). A measure of positive and negative affect for children: Scale development and preliminary validation. *Psychol Assess*, *11*(3), 236–238.

Markowitz, J., Butler, D., Volkening, L., Antisdel, J., Anderson, B., & Laffel, L. (2010). Brief screening tool for disordered eating in diabetes: inter- nal consistency and external validity in a contemporary sample of pediatric patients with type 1 diabetes. *Diabetes Care*, *33*(3), 495–500.

Merell, K. W. (2011). *SEARS*. https://www.parinc.com/Products/Pkey/406

Nabors, L. A., Graves, M. L., Fiser, K. A., & Merianos, A. L. (2021). Family resilience and health among adolescents with asthma only, anxiety only, and comorbid asthma and anxiety. *Journal of Asthma*, *58*(12), 1599–1609. https://doi.org/10.1080/02770903.2020.1817939

Phipps, S., Long, A. M., & Ogden, J. (2007). Benefit finding scale for children: preliminary findings from a childhood cancer population. *Journal of Pediatric Psychology*, *32*(10), 1264–1271.

Prince-Embury, S. (2007). Resiliency Scales for Children and Adolescents: A Profile of Personal Strengths. *Canadian Journal of School Psychology*, *22*(2), 255–261.

Pynoos, R., Rodriquez, N., Steinberg, A., Stuber, M., & Frederick, C. (1998). *The University of California at Los Angeles Posttraumatic Stress Disorder Reaction Index (UCLA-PTSD RI) for DSM-IV (Revision 1)*.

Reynolds, C., & Kamphaus, R. (2004). *Behavior Assessment System for Children Manual* (2nd ed.). AGS Publishing.

Roberts, R., Lewinsohn, P., & Seeley, J. (1993). A brief measure of loneliness suitable for use with adolescents. *Psychol Rep*, *72*, 1379–1391.

Schwartz, L., & Drotar, D. (2009). Health-related hindrance of personal goal pursuit and well-being of young adults with cystic fibrosis, pediatric cancer survivors, and peers without a history of chronic illness. *J Pediatr Psychol*, *34*(9), 954–965.

Thompson, M., & Gray, J. (1995). Development and validation of a new body-image assessment scale. *J Pers Assess*, *64*(2), 258–269.

Varni, J., Seid, M., & Rode, C. (1999). The PedsQL^TM^ measurement model for the pediatric quality of life inventory. *Med Care*, *3*, 126–139.

Varni, J. W., Seid, M., & Kurtin, P. S. (1999). Pediatric health-related quality of life measurement technology: a guide for health care decision makers. *Journal of Clinical Outcomes Management*, *6*, 33–40.

Wagnild, G. M., & Young, H. M. (1993). Development and psychometric evaluation of the Resilience Scale. *Journal of Nursing Measurement*, *1*(2), 165–178.

Welch, G., Jacobson, A., & Polonsky, W. (1997). The problem areas in diabetes scale: An evaluation of its clinical utility. *Diabetes Care*, *20*, 760–766.

Wood, D., Sawicki, G., Miller, M., & et al. (2014). The Transition Readiness Assessment Questionnaire (TRAQ): its factor structure, reliability, and validity. *Acad Pediatr*, *14*, 415–422.

Yang, H., Chen, Y., & Wang, J. (2012). Measuring knowledge of patients with congenital heart disease and their parents: Validity of the ‘Leuven Knowledge Questionnaire for Congenital Heart Disease.’ *Eur J Cardiovasc Nurs*, *11*, 77–84.
