# Supplementary material for: Defining and Measuring Resilience in Children with a Chronic Disease: a Scoping Review
Source: Advers Resil Sci. 2023 Apr 10;4(2):105–23. doi: 10.1007/s42844-023-00092-2 (PMC10088629; doi:10.1007/s42844-023-00092-2)
Supplement: Supplementary file 4 — Supplementary file4 (DOCX 214 KB) [file 42844_2023_92_MOESM4_ESM.docx]

| **Instruments** | **Previously used in youth with diagnosis** | **Items** | **Response** | **Range ^a^** | **Assessment of resilience factors** | | | | | |
| --- | --- | --- | --- | --- | --- | --- | --- | --- | --- | --- |
|  |  |  |  |  | **Internal** | | **Disease** | **External** | | |
|  |  |  |  |  | **Cognitive, social, and emotional competence factors** | | **Disease-related factors** | **Caregiver factors** | **Peer factors** | **Contextual factors** |
| **Adolescent Resilience Questionnaire** (Gartland et al., 2011) | Obesity | 77 | 5-point Likert scale | 77-385 |  | |  |  |  |  |
|  |  |  |  |  | **●**  **●**  **●**  **●**  **●**  ●  ●  ●  ●  ●  ●  ● | Confidence  Emotional insight  Negative cognition  Empathy  Social skills  Family connectedness  Family availability  Peer connectedness  Peer availability  Supportive school environment  School connectedness  Community connectedness | | | | |
| **Antiretroviral treatment (ART) motivation** (Fisher et al., 2006) | HIV/AIDS | NR | 4-point Likert scale | NR |  | |  |  |  |  |
|  |  |  |  |  | ● | Therapy motivation (personal/social) | | | | |
| **Automatic Thoughts Questionnaire (ATQ)** (Hollon & Kendall, 1980) | T1D | 30 | 5-point Likert scale | 30-150 |  | |  |  |  |  |
|  |  |  |  |  | **●**  **●**  **●**  **●** | Personal maladjustment  Self-concepts and expectations  Low self-esteem  Helplessness | | | | |
| **Avoidance and Fusion Questionnaire for Youth (AFQ-Y)** (L. Greco et al., 8 C.E.) | JIA | 17 | 5-point Likert scale | 0-86 |  | |  |  |  |  |
|  |  |  |  |  | **●** | Psychological flexibility | | | | |
| **Beck Self- Concept Inventory (BSCI-Y)** (Cho et al., 2009) | Cancer | 20 | 4-point Likert scale | 0-60 |  | |  |  |  |  |
|  |  |  |  |  | **●**  **●**  **●**  **●** | Positive self-worth  Self-esteem  Potency  Competency | | | | |
| **Child and Adolescent Mindfulness Measure (CAMM)** (L. A. Greco et al., 2011) | Sickle cell disease | 10 | 4-point Likert scale | 0-40 |  | |  |  |  |  |
|  |  |  |  |  | **●** | Mindfulness | | | | |
| **Child Attitude Toward Illness Scale (CATIS)** (Austin & Huberty, 1993) | Food allergy | 13 | 5-point Likert scale | 13-65 |  | |  |  |  |  |
|  |  |  |  |  | ● | Attitudes toward having a condition | | | | |
| **Children’s Attributional Style Questionnaire-Revised (CASQ-R)** (Kaslow et al., 1978) | CHD | 24 | 2-point Likert scale | NR |  | |  |  |  |  |
|  |  |  |  |  | **●** | Attributional style | | | | |
| **Child Behavior Checklist (CBCL)** (Achenbach, 1991) | DMD | 118 | 3-point Likert scale | 0-236 |  | |  |  |  |  |
|  |  |  |  |  | **●**  **●**  **●** | Internalizing behaviors  Externalizing behaviors  Social competence | | | | |
| **Child self-efficacy scale (CSES)** (Bursch et al., 2006) | Gastrointestinal disorders | 7 | 5-point Likert Scale | 7-35 |  | |  |  |  |  |
|  |  |  |  |  | ● | Self-efficacy despite pain | | | | |
| **Connor-Davidson resiliency questionnaire (CD-RISC-10/25)** (Campbell-Sills & Stein, 2007; Connor & Davidson, 2003) | IBD, cancer, T1D | 10  25 | 5-point Likert scale | 0-40  0-100 |  | |  |  |  |  |
|  |  |  |  |  | **●**  **●**  **●** | Perceived ability to tolerate experiences  Humor  Self-efficacy | | | | |
| **Coping Efficacy Questionnaire (CEQ)** (Hood et al., 2018; I. N. Sandler et al., 2000) | T1D | 7 | 4-point Likert scale | NR |  | |  |  |  |  |
|  |  |  |  |  | **●** | Coping skills | | | | |
| **Children’s Hope Scale (CHS)** (Snyder et al., 1997) | SCD | 6 | 6-point Likert scale | 6-36 |  | |  |  |  |  |
|  |  |  |  |  | **●** | Agency as related to achieving goals | | | | |
| **Childrearing behavior Questionnaire** (Park, 1995) | Atopic dermatitis | 30 | 4-point Likert scale | 30-120 |  | |  |  |  |  |
|  |  |  |  |  | ●  ●  ● | Warmth acceptance  Rejection-restriction  Permissiveness non-intervention | | | | |
| **Coping Orientation to Problems Experienced (COPE)** (Carver, 1997) | T1D | 28 | 4-point Likert scale | 28-112 |  | |  |  |  |  |
|  |  |  |  |  | **●** | Coping (problem-focused, emotion-focused, avoidant) | | | | |
| **Coping Inventory for Stressful Situations (CISS)**(Endler & Parker, 1999) | CHD | 48 | 5-point Likert scale | 48-240 |  | |  |  |  |  |
|  |  |  |  |  | **●** | Coping (task-oriented, emotion-oriented, avoidance oriented) | | | | |
| **Chronic Pain Acceptance Questionnaire (CPAQA)** (McCracken et al., 2010) | JIA, SCD | 20 | 5-point Likert scale | 0-80 |  | |  |  |  |  |
|  |  |  |  |  | **●** | Pain acceptance | | | | |
| **Coping Strategies Questionnaire for Sickle Cell Disease (CSQ)** (Gil et al., 1991) | SCD | 80 | 7-point Likert scale | 0-480 |  | |  |  |  |  |
|  |  |  |  |  | ● | Coping with sickle cell disease-related pain | | | | |
| **Child and Youth Resilience Measures-28 (CYRM-28)** (Liebenberg et al., 2012) | MS | 28 | 5-point Likert scale | 28-140 |  | |  |  |  |  |
|  |  |  |  |  | **●**  **●**  ●  ●  ●  ●  ●  ● | Personal skills  Social skills  Physical caregiving  Psychological caregiving  Peer support  Spiritual context  Educational context  Cultural context | | | | |
| **Coping Efficacy Scale (CSE)** (I. Sandler et al., 2000) | T1D | 8 | 4-point Likert scale | 8-32 |  | |  |  |  |  |
|  |  |  |  |  | **●** | Perceived ability to cope with stress | | | | |
| **Diabetes Family Conflict Scale-Revised (DFCS-R)** (Hood et al., 2007) | T1D | 19 | 3-point Likert scale | 19-57 |  | |  |  |  |  |
|  |  |  |  |  | ● | Diabetes-related family conflict | | | | |
| **Diabetes Strengths and Resilience measure for adolescents (DSTAR-teen)** (Hilliard et al., 2017) | T1D | 12 | 5-point Likert scale | 12-60 |  | |  |  |  |  |
|  |  |  |  |  | ●  ● | Managing and adapting to illness  Availability and support from others | | | | |
| **Emotional Approach & Coping Scale (EAC)** (Huston et al., 2016) | T1D | 16 | 4-point Likert scale | 4-64 |  | |  |  |  |  |
|  |  |  |  |  | **●**  **●** | Emotional processing  Emotion expression | | | | |
| **Ego-Resiliency Scale** (Block & Block, 1980; Prince-Embury, 2013) | MS | 14 | 4-point Likert scale | 14-56 |  | |  |  |  |  |
|  |  |  |  |  | **●** | Adaptive flexibility | | | | |
| **Family Adaptability and Cohesion Scale (FACES II/III)** (Lim et al., 1990) | Cancer | 20 | 5-point Likert scale | 10-50 |  | |  |  |  |  |
|  |  |  |  |  | ●  ● | Family cohesion  Family adaptability | | | | |
| **Family Resilience Assessment scale (FRAS-C)** (Li et al., 2016) | Epilepsy | 32 | 4-point Likert scale | 32-128 |  | |  |  |  |  |
|  |  |  |  |  | **●**  ●  ● | Maintaining a positive outlook  Family communication and problem solving  Utilizing social resources | | | | |
| **Family Strengths Scale** (Olson, 1985) | Cancer | 12 | 5-point Likert scale | 12-60 |  | |  |  |  |  |
|  |  |  |  |  | ●  ●  ●  ●  ● | Interactional patterns  Family values  Family coping strategies  Family commitment  Resource mobilization skills | | | | |
| **Haase Adolescent Resilience in Illness Scale (HARIS)** (Haase et al., 2014) | Cancer, CHD, HSCT | 13 | 6-point Likert scale | 13-78 |  | |  |  |  |  |
|  |  |  |  |  | **●**  **●**  **●** | Self-esteem  Sense of mastery  Positive expectation | | | | |
| **Herth Hope Index** (Herth, 1992) | Cancer | 4  4 | 5-point Likert scale | 1-20  1-20 |  | |  |  |  |  |
|  |  |  |  |  | **●**  **●**  **●** | Hope-derived meaning  Positive readiness and expectancy  Sense of interconnectedness | | | | |
| **Hemingway Measure of Adolescent Connectedness (HMAC)** (Karcher, 2005; Karcher & Sass, 2010) | Cancer | 57 | 5-point Likert scale | 57-285 |  | |  |  |  |  |
|  |  |  |  |  | **●**  ●  ●  ● | Self-connection  Family connectedness  Peer connectedness  School connectedness | | | | |
| **Healthy Kids Resilience Assessment Module (HKRM)** (Martins, 2005) | T1D, allergic or neurological diseases | 18 | 4-point Likert scale | 18-72 |  | |  |  |  |  |
|  |  |  |  |  | **●**  **●**  **●**  **●**  **●**  **●** | Achievement motivation  Communication  Empathy  Problem-solving  Self-efficacy  Self-awareness | | | | |
| **Hopelessness Scale for Children (HSC)** (Kazdin et al., 1986) | T1D | 17 | True/false items | 0-17 |  | |  |  |  |  |
|  |  |  |  |  | **●** | Negative expectations about the future | | | | |
| **Illness Cognition Questionnaire (ICQ)** (Evers & Kraaimaat, 2009) | Leukemia | 18 | 4-point Likert scale | 4-72 |  | |  |  |  |  |
|  |  |  |  |  | ●  ●  ● | Helplessness  Acceptance  Perceived benefit | | | | |
| **Jalowiec Coping Scale-Revised** (Jalowiec, 1984) | Cancer | 32 | 4-point Likert scale | 0-96 |  | |  |  |  |  |
|  |  |  |  |  | **●** | Coping (emotive, evasive, confrontive, optimistic, supporting) | | | | |
| **Kessler-6 psychological distress scale (K6)** (Kessler et al., 2010) | Cancer | 6 | 5-point Likert scale | 6-30 |  | |  |  |  |  |
|  |  |  |  |  | **●** | Psychological distress | | | | |
| **Life Orientation Test (LOT)** (Scheier & Carver, 1987) | T1D | 8 | 5-point Likert scale | 0-40 |  | |  |  |  |  |
|  |  |  |  |  | **●**  **●** | Optimism  Confidence in one’s own ability | | | | |
| **McCorkcle Symptom Distress Scale** (McCorkle, 1987) | Cancer | 11 | 5-point Likert scale | 11-55 |  | |  |  |  |  |
|  |  |  |  |  | ● | Symptom distress | | | | |
| **Mishel Uncertainty in Illness Scale** (Mishel, 1982) | Cancer | 28 | 5-point Likert scale | 28-140 |  | |  |  |  |  |
|  |  |  |  |  | ● | Uncertainty in illness | | | | |
| **Parent-Adolescent Communication Scale** (Olson, 1985) | Cancer | 20 | 5-point Likert scale | 20-100 |  | |  |  |  |  |
|  |  |  |  |  | ●  ● | Open communication  Communication problems | | | | |
| **Pediatric Quality of Life Inventory (PedsQL)** (Varni et al., 1999) | T1D | 23 | 5-point Likert scale | 0-92 |  | |  |  |  |  |
|  |  |  |  |  | **●**  **●**  ●  ● | Physical functioning  Emotional functioning  Social functioning  School-related functioning | | | | |
| **Pediatric Quality of Life Inventory (PedsQL), diabetes module** (Varni et al., 1999) | T1D | 33 | 5-point Likert scale | 0-132 |  | |  |  |  |  |
|  |  |  |  |  | ● | Disease-related quality of life | | | | |
| **Patient-Based Assessment and Counseling for Physical Activity and Nutrition-Adolescent assessment forms (PACE-Adolescent)** (Hagler et al., 2006; Prochaska et al., 2001; Prochaska & Sallis, 2004) | T2D | NR | 5-point Likert scale | NR |  | |  |  |  |  |
|  |  |  |  |  | **●** | Readiness for behavior change | | | | |
| **Pediatric Cancer Coping Scale (PCCS)** (Wu et al., 2011) | Cancer | 33 | 3-point Likert scale | 0-66 |  | |  |  |  |  |
|  |  |  |  |  | **●** | Coping style (cognitive, problem-oriented, and defensive) | | | | |
| **Perceived social support** (Procidano & Heller, 1983) | Cancer | 60 | 5-point Likert scale | NR |  | |  |  |  |  |
|  |  |  |  |  | ●  ●  ● | Perceived social support from healthcare providers  Perceived social support from family  Perceived social support from friends | | | | |
| **Personal Relationship Measurement** (J. Kim, 1992) | Cancer | 14 | 5-point Likert scale | NR |  | |  |  |  |  |
|  |  |  |  |  | ● | Friendship | | | | |
| **Peabody Picture Vocabulary Test—Third Edition (PPVT-III)** (Dunn & Dunn, 2007) | DMD | 204 | 4-point Likert scale | NR |  | |  |  |  |  |
|  |  |  |  |  | **●** | Verbal intelligence | | | | |
| **Quick Big Five** (Vermulst & Geriis, 2005) | CHD | 30 | 7-point Likert scale | NR |  | |  |  |  |  |
|  |  |  |  |  | **●** | Personality type (extraversion, agreeableness, conscientiousness, emotional stability, openness) | | | | |
| **Reed Spiritual Perspective Scale** (Reed, 1987) | Cancer | 10 | 6-point Likert scale | 10-60 |  | |  |  |  |  |
|  |  |  |  |  | ● | Spiritual frequency and beliefs | | | | |
| **Resilience measurement instrument for children with chronic illness** (D. H. Kim & Yoo, 2010) | Atopic dermatitis, cancer | 32 | 4-point Likert scale | 32-128 |  | |  |  |  |  |
|  |  |  |  |  | **●**  **●**  **●**  ●  ● | Self-understanding  Self-reliance  Resourcefulness  Family relationships  Interpersonal intimacy | | | | |
| **Resilience Scale for Children and Adolescents (RSCA)** (Prince-Embury, 2007) | T1D, Orofacial anomalies | 64 | 5-point Likert scale | 0-256 |  | |  |  |  |  |
|  |  |  |  |  | **●**  **●**  **●** | Self-perception of skills and competences  Emotional reactivity  Sense of relatedness | | | | |
| **Resilience Assessment Scale for Children and Adolescents (SPP-18)** (Ogińska-Bulik & Juczyński, 2011) | Liver or renal transplantation | 18 | 5-point Likert scale | 0-72 |  | |  |  |  |  |
|  |  |  |  |  | **●**  **●**  **●** | Sense of mastery  Sense of relatedness  Emotional reactivity | | | | |
| **Resilience Scale (RS), Wagnild and Young** (Wagnild & Young, 1993a) | CHD, CKD, T1D, cancer | 25 | 7-point Likert scale | 25-175 |  | |  |  |  |  |
|  |  |  |  |  | **●**  **●**  **●**  **●**  **●** | Purpose  Perseverance  Self-reliance  Equanimity  Authenticity | | | | |
| **Resilience Scale, 14-item (RS-14)** (Wagnild & Young, 1993b) | Cancer | 10 | 4-point Likert scale | 10-40 |  | |  |  |  |  |
|  |  |  |  |  | **●**  **●**  **●**  **●**  **●** | Sense of purpose and meaning  Authenticity  Equanimity  Self‐reliance  Perseverance | | | | |
| **Responses to Stress Questionnaire (RSQ)** (Connor-Smith et al., 2000) | T1D | 57 | 4-point Likert scale | 57-171 |  | |  |  |  |  |
|  |  |  |  |  | ●  ● | Coping strategies in response to illness-related stressors  Involuntary stress response in response to illness-related stressors | | | | |
| **Rosenberg Self-Esteem Scale** (Rosenberg, 1979) | Cancer, T1D | 10 | 4-point Likert scale | NR |  | |  |  |  |  |
|  |  |  |  |  | **●**  **●** | Self-esteem  Self-worth/self-acceptance | | | | |
| **School Connectedness Scale (SCS)** (McNeely et al., 2002; Resnick et al., 1997) | HIV/AIDS | 6 | 4-point Likert scale | 6-24 |  | |  |  |  |  |
|  |  |  |  |  | ●  ●  ● | Connectedness to peers  Connectedness to the school  Connectedness to adults in schools | | | | |
| **Self-efficacy for antiretroviral treatment use** (Fisher et al., 2006) | HIV/AIDS | NR | 5-point Likert scale | NR |  | |  |  |  |  |
|  |  |  |  |  | ● | Self-efficacy for medication | | | | |
| **Self-efficacy for diabetes (SED)** (Grossman et al., 1987) | T1D | 35 | 6-point Likert scale | 35-175 |  | |  |  |  |  |
|  |  |  |  |  | ● | Self-efficacy in relation to diabetes management | | | | |
| **Social support** (Cutrona & Russel, 1987) | HIV/AIDs | 6 | 4-point Likert scale | NR |  | |  |  |  |  |
|  |  |  |  |  | ● | Social support promoting therapy adherence | | | | |
| **Social Problem-Solving Inventory-Revised short form (SPSI-R:S)** (Maydeu-Olivares & D’Zurilla, 1996; Weissberg-Benchell et al., 2016) | T1D | 25 | 5-point Likert scale | 0-100 |  | |  |  |  |  |
|  |  |  |  |  | **●** | Problem solving | | | | |
| **School Support Scale (SSS)** (Hanson & Kim, 2007) | HIV/AIDS | 5 | 5-point Likert scale | 5-25 |  | |  |  |  |  |
|  |  |  |  |  | ● | School liking | | | | |
| **Scale of Satisfaction with Social Support (SSSS)** (Gaspar et al., 2009; Ribeiro, 1999) | T1D, allergic or neurological diseases | 12 | 5-point Likert scale | 18-72 |  | |  |  |  |  |
|  |  |  |  |  | ●  ● | Satisfaction with social support  Activities connected to social support | | | | |
| **School Adjustment Test** (Im, 1993) | Cancer | 8 | 5-point Likert scale | NR |  | |  |  |  |  |
|  |  |  |  |  | ● | Relationship with teachers | | | | |
| **The Neil and Dias Resilience scale** (Neill & Dias, 2001) | T1D | 15 | 7-point Likert scale | 15-105 |  | |  |  |  |  |
|  |  |  |  |  | **●**  **●** | Personal competence  Acceptance | | | | |
| **Youth Self Report (YSR)** (Achenbach & Rescorla, 2001) | T1D | 20 | 3-point Likert scale | 0-40 |  | |  |  |  |  |
|  |  |  |  |  | **●**  **●** | Perception of competence  Perception of social competence | | | | |
| **7Cs Tool** (Barger et al., 2017) | Obesity | 7 | 3-point Likert scale | 0-2 |  | |  |  |  |  |
|  |  |  |  |  | **●**  **●**  **●**  **●**  **●**  **●**  **●** | Competence  Confidence  Character  Connection  Caring  Coping  Control | | | | |

|  | Disease | CHD = Congenital Heart Disease; CKD = Chronic Kidney Disease; DMD = Duchenne Muscular Dystrophy; HIV = Human Immunodeficiency Virus; IBD = Inflammatory Bowel Diseases; JIA = juvenile idiopathic arthritis; MS = Multiple Sclerosis; SCD = Sickle Cell Disease; T1D = Type 1 diabetes; T2D = Type 2 diabetes. |
| --- | --- | --- |
| **Legend** |  |  |
|  | Other | NA = not applicable; NR = not reported |
|  |  |  |

Achenbach, T. M. (1991). *The Manual for the Child Behavior Checklist/4-18 and 1991 Profile*. University of Vermont, Department of Psychiatry.

Achenbach, T. M., & Rescorla, L. A. (2001). *Manual for the ASEBA School-Age Forms and Profiles*. University of Vermont, Research Centre for Children, Youth and Families.

Austin, J. K., & Huberty, T. J. (1993). Development of the Child Attitude Toward Illness Scale. *J Pediatr Psychol*, *18*, 467–480.

Barger, J., Vitale, P., Gaighan, J., & Feldman-Winter, L. (2017). Measuring resilience in the adolescent population: a succinct tool for outpatient adolescent health. *J Pediatr2*, *189*, 201–206.

Block, J. H., & Block, J. (1980). *The role of ego-control and Ego- resiliency in the origination of behavior* (Vol. 13). WA Collings (red.). The Minnesota Symposia on Child Psychology.

Bursch, B., Tsao, J. C. I., Meldrum, M., & Zeltzer, L. K. (2006). Preliminary validation of a self-efficacy scale for child functioning despite chronic pain (child and parent versions). *Pain*, *125*(1), 35–42. https://doi.org/10.1016/j.pain.2006.04.026

Campbell-Sills, L., & Stein, M. B. (2007). Psychometric analysis and refinement of the connor–davidson resilience scale (CD-RISC): Validation of a 10-item measure of resilience. *Journal of Traumatic Stress*, *20*(6), 1019–1028.

Carver, C. S. (1997). You want to measure coping but your protocol’s too long: Consider the Brief COPE. *International Journal of Behavioral Medicine*, *4*, 92–100.

Cho, S., Hung, L., Su, C., & Chen, H. (2009). A research of the Chinese version Beck Youth Inventories. *Psychol Test*, *56*(4).

Connor, K. M., & Davidson, J. R. T. (2003). Development of a new Resilience scale: The Connor-Davidson Resilience scale (CD-RISC). *Depression and Anxiety*, *18*(2), 76–82.

Connor-Smith, J. K., Compas, B. E., Wadsworth, M. E., & al, E. (2000). Responses to stress in adolescence: measurement of coping and involuntary stress responses. *Journal of Consulting and Clinical Psychology*, *68*, 976–992.

Cutrona, C. E., & Russel, D. W. (1987). The provisions of social relationships and adaptation to stress. *Advances in Personal Relationships*, *1*(37–67).

Dunn, L. M., & Dunn, D. M. (2007). *Peabody Picture Vocabulary Test* (Fourth edi). Pearson Assessments.

Endler, N., & Parker, J. (1999). *Coping Inventory for Stressful Situations (CISS): Manual* (2nd ed.). Multi-Health Systems.

Evers, A., & Kraaimaat, F. (2009). *Illness Cognition Questionnaire*. Www.Andreaevers.Nl. https://www.andreaevers.nl/uploads/bestanden/ICQ18-En.pdf

Fisher, J. D., Fisher, W. A., Amico, K. R., & et al. (2006). An information-motivation-behavioral skills model of adherence to antiretroviral therapy. *Health Psychology*, *25*(4), 462–473.

Gartland, D., Bond, L., Olsson, C. A., Buzwell, S., & Sawyer, S. M. (2011). Development of a multi-dimensional measure of resilience in adolescents: the Adolescent Resilience Questionnaire. *BMC Medical Research Methodology*, *11*(1), 134. https://doi.org/10.1186/1471-2288-11-134

Gaspar, T., Robeiro, J., Matos, M., Leal, I., & Ferreira, A. (2009). Psychometric Properties of a Brief Version of the Escala de Satisfação com o Suporte Social for Children and Adolescents. *Span J Psychol*, *12*(1), 360–372.

Gil, K. M., Williams, D. A., Thompson, R. J., & et al. (1991). Sickle Cell Disease in Children and Adolescents: The Relation of Child and Parent Pain Coping Strategies to Adjustment. *Journal of Pediatric Psychology*, *16*(5), 643–663.

Greco, L. A., Baer, R. A., & Smith, G. T. (2011). Assessing mindfulness in children and adolescents: Development and validation of the Child and Adolescent Mindfulness Measure (CAMM). *Psychological Assessment*, *23*(3), 606–614. https://doi.org/10.1037/a0022819

Greco, L., Lambert, W., & Baer, R. (8 C.E.). Psychological inflexibility in childhood and adolescence: Development and evaluation of the avoidance and fusion questionnaire for youth. *Psychol Assess2*, *20*(2), 93–102.

Grossman, H. Y., Brink, S., & Hauser, S. T. (1987). Self-efficacy in adolescent girls and boys with insulin-dependent diabetes mellitus. *Diabetes Care*, *10*(3), 324–329.

Haase, J. E., Kintner, E. K., Monahan, P. O., & Robb, S. L. (2014). The resilience in illness model, part 1: exploratory evaluation in adolescents and young adults with cancer. *Cancer Nursing*, *37*(3), E1-12.

Hagler, A., Calfas, K., Norman, G., Sallis, J., & Patrick, K. (2006). Construct validity of physical activity and sedentary behaviors staging measures for adolescents. *Ann Behav Med*, *31*, 186–193.

Hanson, T. L., & Kim, J. O. (2007). *Measuring resilience and youth development: The psychometric properties of the Healthy Kids Survey*.

Herth, K. (1992). Abbreviated instrument to measure hope: development and psychometric evaluation. *J Adv Nurs*, *17*, 1251–1259.

Hilliard, M. E., Iturralde, E., Weissberg-Benchell, J., & Hood, K. K. (2017). The Diabetes Strengths and Resilience Measure for Adolescents With Type 1 Diabetes (DSTAR-Teen): Validation of a New, Brief Self-Report Measure. *Journal of Pediatric Psychology*, *42*(9), 995–1005.

Hollon, S. D., & Kendall, P. C. (1980). Cognitive self-statements in depression: Development of an automatic thoughts questionnaire. In *Cognitive Therapy and Research* (Vol. 4, Issue 4, pp. 383–395). https://doi.org/10.1007/BF01178214

Hood, K. K., Butler, D. A., Anderson, B. J., & Laffel, L. M. B. (2007). Updated and Revised Diabetes Family Conflict Scale. *Diabetes Care*, *30*(7), 1764–1769. https://doi.org/10.2337/dc06-2358

Hood, K. K., Iturralde, E., Rausch, J., & et al. (2018). Preventing diabetes distress in adolescents with type 1 diabetes: Results 1 year after participation in the STePS program. *Diabetes Care*, *41*(8), 1623–1630.

Huston, S. A., Bloun, R. L., & Heidsec, T. (2016). Resilience, emotion processing and emotionexpression among youth with type 1 diabetes. *Pediatric Diabetes*, *17*, 623–631.

Im, J. (1993). *The relationships between dependency and school adjustment of children (master thesis)*. Korea National University of Education.

Jalowiec, A. (1984). Psychometric assessment of the Jalowiec Coping Scale. *Nurs Res*, *33*, 157–161.

Karcher, M. J. (2005). *The Hemingway: Measure of Adolescent Connectedness: A manual for scoring and in- terpretation. Unpublished Manuscript*. University of Texas.

Karcher, M. J., & Sass, D. (2010). A multicultural assessment of adolescent connectedness: Testing measurement invariance across gender and ethnicity. *Journal of Counseling Psychology*, *57*, 274–289.

Kaslow, N. J., Tannenbaum, R. L., & Seligman, M. E. P. (1978). *The KASTAN: A children’s attributional style questionnaire*.

Kazdin, A. E., Rodgers, A., & Colbus, D. (1986). The Hopelessness Scale for Children: Psychometric characteristics and concurrent validity. *Journal of Consulting and Clinical Psychology*, *54*(2), 241–245. https://doi.org/10.1037/0022-006X.54.2.241

Kessler, R., Green, J., Gruber, M., & et al. (2010). Screening for serious mental illness in the general population with the K6 screening scale: results from the WHO World Mental Health (WMH) survey initiative. *Int J Methods Psychiatr Res*, *19*, 4–22.

Kim, D. H., & Yoo, I. Y. (2010). Development of a Questionnaire to Measure Resilience in Children with Chronic Diseases. *Journal of Korean Academy of Nursing*, *40*(2), 236.

Kim, J. (1992). The relation between daily stress and emotional experience on the adjustment of middle-aged women: impacts of psychological and social resources. *J. Korean Psychol. Assoc*, *4*, 54–68.

Li, Y., Lu, P. W., & Sun, J. (2016). Research on post-traumatic growth status and influencing factors of adolescent patients with chronic diseases. *Journal of Chinese Nursing Management*, *7*, 914–919.

Liebenberg, L., Ungar, M., & van d.V.jver, F. (2012). Validation of the Child and Youth Resilience Measure-28 (CYRM-28) Among Canadian Youth. *Research on Social Work Practice*, *22*(2), 219–226.

Lim, J., Lee, K., Oh, M., Kwak, K., Lee, H., & Yoon, B. (1990). A study on reliability and validity of FACES. *J. Korean Acad. Fam. Med*, *11*, 8–17.

Martins, M. H. (2005). *Contribuições para a análise de crianças e jovens em situação de risco - Resiliência e Desenvolvimento [Contributions for the analysis of children and adolescentes in risky situations – resilience and development]*. Universidade do Algarve.

Maydeu-Olivares, A., & D’Zurilla, T. (1996). A factor analytic study of the social problem-solving inventory: an integration of theory and data. *Cognitive Therapy and Research*, *20*, 115–133.

McCorkle, R. (1987). The measurement of symptom distress. *Semin Oncol Nurs*, *3*, 248–256.

McCracken, L., Gauntlett-Gilbert, J., & Eccleston, C. (2010). Acceptance of pain in adolescents with chronic pain: Validation of an adapted assessment instrument and preliminary correlation analyses. *Eur J Pain*, *14*(3), 316–320.

McNeely, C., Nonnemaker, J., Blum, R., & al, E. (2002). Promoting school connectedness: Evidence from the National Longitudinal Study of Adolescent Health. *Journal of School Health*, *72*, 138–146.

Mishel, M. (1982). The measurement of uncertainty in illness. *Nurs Res*, *30*, 258–263.

Neill, J. T., & Dias, K. L. (2001). Adventure education and resilience: The double-edged sword. *Journal of Adventure Education & Outdoor Learning*, *1*(2), 35–42.

Ogińska-Bulik, N., & Juczyński, Z. (2011). “Prężność u dzieci i młodzieży: charakterystyka i pomiar–polska skala SPP-18.” *Polskie Forum Psychologiczne*, *16*(1).

Olson, D. (1985). *Family inventories*. Family Social Science, University of Minnesota.

Park, Y. (1995). *The Relationships between Parental Childrearing Behaviors and Sibling Relations and Children’s Self- esteem*.

Prince-Embury, S. (2007). Resiliency Scales for Children and Adolescents: A Profile of Personal Strengths. *Canadian Journal of School Psychology*, *22*(2), 255–261.

Prince-Embury, S. (2013). The Ego-Resiliency Scale by Block and Kremen (1996) and Trait Ego-Resiliency. In *Prince-Embury S., Saklofske D. (eds) Resilience in Children, Adolescents, and Adults*. Springer.

Prochaska, J., & Sallis, J. (2004). Reliability and validity of a fruit and vegetable screening measure for adolescents. *Adoles Health*, *33*, 163–165.

Prochaska, J., Sallis, J., & Long, B. (2001). A physical activity screening measure for use with adolescents in primary care. *Arch Adolesc Med*, *155*, 554–559.

Procidano, M., & Heller, K. (1983). Measures of perceived social support from friends and from family: three validation studies. *Am J Community Psychol*, *11*(1–24).

Reed, P. G. (1987). Spirituality and well-being in terminally ill hospitalized adults. *Research in Nursing Health*, *10*(5), 344–355.

Resnick, M. D., Bearman, P. S., Blum, R., & al, E. (1997). Protecting adolescents from harm: Findings from the National Longitudinal Study on Adolescent Health. *Journal of American Medical Association*, 823–832.

Ribeiro, J. (1999). Escala de Satisfação com o Suporte Social (ESSS) [Satisfaction with Social Support Scale]. *Analise Psicologica*, *3*(17), 547–558.

Rosenberg, M. (1979). *Conceiving the Self*. Basic Books.

Sandler, I. N., Tein, J. Y., Wolchik, S., & al, E. (2000). Coping Efficacy and Psychological Problems of Children of Divorce. *Child Development*, *71*(4), 1099–1118.

Sandler, I., Tein, J., Wolchik, S., & et al. (2000). Coping Efficacy and Psychological Problems of Children of Divorce. *Child Development*, *71*(4), 1099–1118.

Scheier, M., & Carver, C. (1987). Dispositional optimism and physical well-being: The influence of generalized outcome expectancies on health. *Journal of Personality*, *55*(2), 169–210.

Snyder, C. R., Hoza, B., Pelham, W. E., Rapoff, M., Ware, L., Danovsky, M., Highberger, L., Ribinstein, H., & Stahl, K. J. (1997). The Development and Validation of the Children’s Hope Scale. *Journal of Pediatric Psychology*, *22*(3), 399–421.

Varni, J. W., Seid, M., & Kurtin, P. S. (1999). Pediatric health-related quality of life measurement technology: a guide for health care decision makers. *Journal of Clinical Outcomes Management*, *6*, 33–40.

Vermulst, A. A., & Geriis, J. R. M. (2005). *QBF: Quick Big Five persoonlijkheidstest handleiding [Quick Big Five personality test manual]* (LDC Public).

Wagnild, G. M., & Young, H. M. (1993a). Development and psychometric evaluation of the Resilience Scale. *Journal of Nursing Measurement*, *1*(2), 165–178.

Wagnild, G. M., & Young, H. M. (1993b). Development and psychometric evaluation of the Resilience Scale. *Journal of Nursing Measurement*, *1*(2), 165–178.

Weissberg-Benchell, J., Rausch, J., Iturralde, E., & et al. (2016). A randomized clinical trial aimed at preventing poor psychosocial and glycemic outcomes in teens with type 1 diabetes (T1D). *Contemporary Clinical Trials*, *49*(24), 78–84.

Wu, L., Chin, C., Chen, C., Lai, F., & Tseng, Y. (2011). Development and validation of the paediatric cancer coping scale. *Journal of Advanced Nursing*, *67*(5), 1142–1152.
